# Untargeted Metabolomics Reveals Metabolic Perturbations in Community-Dwelling Elderly Exposed to PM_2.5_-Bound Ester Compounds

**DOI:** 10.3390/metabo16080553

**Published:** 2026-08-05

**Authors:** Shilin Chen, Ruoyu Li, Wenli Wang, Dan Wang, Yuling Zhang, Yongxin Wang, Haoneng Hu, Jianjun Xiang, Yu Jiang, Chuancheng Wu

**Affiliations:** 1 Department of Preventive Medicine, School of Public Health, Fujian Medical University, Fuzhou 350108, China; 2 The Key Laboratory of Environment and Health, School of Public Health, Fujian Medical University, Fuzhou 350108, China

**Keywords:** fine particulate matter, ester compounds, untargeted metabolomics

## Abstract

**Background/Objectives**: Ambient fine particulate matter (PM_2.5_) poses significant health risks to older adult populations, yet the specific contributions of its chemical constituents, particularly non-phthalate and non-organophosphate ester compounds, remain poorly understood. This study aimed to elucidate the mechanistic links between PM_2.5_-bound ester exposures and metabolic pathway alterations in elderly individuals. **Methods**: A total of 258 elderly residents aged 60 years or older from Fuzhou, China, were recruited. Personal PM_2.5_ exposure was monitored over 72 h using UPAS V2 samplers, with chemical components analyzed via gas chromatography–mass spectrometry (GC–MS). Plasma metabolomic profiling was conducted using liquid chromatography–mass spectrometry (LC–MS), and metabolic pathway enrichment was performed using MetaboAnalyst 5.0. Linear regression models adjusted for covariates (age, sex, BMI, lifestyle factors) assessed associations between ester exposures and metabolite abundance. **Results**: The mean PM_2.5_ concentration was 38.06 μg/m^3^, with ester compounds dominating the chemical composition. Twenty high-concentration non-target esters were prioritized for analysis. PM_2.5_ ester exposure was associated with alterations in key metabolic pathways, including steroid biosynthesis, glycolysis/gluconeogenesis, glycerophospholipid metabolism, and purine/pyrimidine metabolism. When interpreted alongside prior epidemiological evidence, these alterations represent putative links to increased risks of insulin resistance, cardiovascular dysfunction, and metabolic syndrome—relationships that require confirmation in prospective cohort studies and controlled toxicological experiments. **Conclusions**: Putatively annotated PM_2.5_-bound ester compounds, particularly non-regulated subclasses, are associated with systemic metabolic alterations in older adults, coincident with perturbations in steroid and lipid metabolism. While these findings are exploratory and hypothesis-generating, they highlight the need to incorporate specific ester profiles into PM_2.5_ risk assessments and develop targeted interventions for vulnerable aging populations.

## 1. Introduction

Ambient fine particulate matter (PM_2.5_), defined as airborne particles with an aerodynamic diameter of 2.5 micrometers or less, represents a pervasive environmental stressor with profound implications for global public health. Due to their minute size and substantial specific surface area, PM_2.5_ particles serve as effective carriers for adsorbed toxic and harmful substances, enabling deep penetration into the respiratory tract where they deposit in alveolar regions [1]. The predominant policy and epidemiology literature still frames PM_2.5_ as a unitary mass metric, yet it is increasingly clear that the health burden is not carried by particle mass alone but by its chemical constitution, which is a complex, spatially variable mixture of elemental carbon, secondary inorganic ions, transition metals, and, critically, condensed or surface-adsorbed organic compounds that ride the particle phase.

Among the organic fraction, ester-containing compounds constitute a chemically diverse and ubiquitously detected class on PM_2.5_ [2], from industrial and consumer chemicals and atmospheric secondary formation, entering the aerosol phase via volatilization or physical processes [3,4]. High-throughput screening routinely detects dozens to thousands of ester-like features in PM_2.5_ extracts, including adipates, benzoates, aliphatic dicarboxylic acid diesters, and specialty plasticizer/UV-absorber esters, most of which lie outside the canonical regulatory focus on ortho-phthalates and organophosphate ester flame retardants [2,5]. Despite structural heterogeneity, they share a reactive ester bond. Experimental evidence shows that their hydrolysis or esterase-mediated biotransformation yields alcohols and carboxylic acids, some acting as mitochondrial uncouplers, electrophilic intermediates, or endocrine disruptors that perturb nuclear receptor signaling and calcium homeostasis [6,7,8]. At the particle–lung interface, co-deposition with redox-active metals and semi-volatile organics creates combinatorial exposure scenarios: particle-derived oxidative stress drives epithelial barrier disruption and dendritic cell activation [9], while locally or systemically generated ester metabolites amplify oxidative injury, parasympathetic dysregulation, and chronic inflammation [10]. Furthermore, emerging evidence links ester species to glycolipid metabolic alterations [11,12,13], supporting their potential role in diabetes and obesity pathogenesis [14,15].

This gap is particularly salient for older adults, who constitute both the fastest-growing global demographic [16] and the population with the highest PM_2.5_-attributable disease burden [6,17]. Aging confers converging vulnerabilities: diminished mucociliary clearance [18] and altered lung geometry prolong particle [19] and sorbed-chemical residence time in the airways [19]; immunosenescence shifts the cytokine milieu toward a pro-inflammatory “inflammaging” baseline [20], lowering the threshold for pollutant-triggered clinical decompensation [21]; and high prevalences of hypertension, metabolic syndrome, and CKD create effect modification that may magnify cardiovascular and renal responses [22,23]. When PM_2.5_ carries putatively bioactive ester constituents, the effective systemic dose depends not only on ambient concentration but also on source profiles, particle organic content, and individual esterase phenotypes, which are variables unmeasured by standard PM_2.5_ mass monitoring.

This study explores potential associations between putatively annotated PM_2.5_-bound ester exposures and metabolic alterations in community-dwelling older adults. Integrating personal exposure monitoring, untargeted metabolomics, and chemical profiling, we characterize metabolic signatures that may inform physiological dysregulation in aging populations. These findings may help guide targeted public health interventions for pollution-vulnerable older adults, pending validation in prospective and experimental studies.

## 2. Materials and Methods

### 2.1. Experimental Instruments

Ultrasonic Personal Air Sampler V2 (UPAS V2; Access Sensor Technologies, Fort Collins, CO, USA), Agilent 7890A gas chromatograph coupled with an Agilent 7250 quadrupole time-of-flight mass spectrometer (GC-Q-TOF or GC/MS; Agilent, Santa Clara, CA, USA), Micropipette (Eppendorf, Hauppauge, NY, USA), MILLIPORE Synergy UV-R Ultrapure Water System (Merck, Darmstadt, Germany), KQ-250DE Digital Ultrasonic Cleaner (Kunshan, China), KS 260 Platform Shaker (IKA, Staufen, Germany), MS205DU Analytical Balance (1/10,000–1/100,000) (METTLER TOLEDO, Greifensee, Switzerland), Digital Display Drying Oven (Boxun Medical Equipment Factory, Shanghai, China), H1650-W Bench-top Micro High-Speed Centrifuge (Xiangyi Centrifuge Instrument Co., Ltd., Changsha, China), Agilent LC-MS System (Agilent, Santa Clara, CA, USA), and Vortex Mixer (Qilin Medical Instrument Factory, Nantong, China), R version 4.3.0 (R Foundation for Statistical Computing, Vienna, Austria), MassHunter Workstation Software v10.0 (Agilent, Santa Clara, CA, USA), EpiData software version 3.1 (EpiData Association, Odense, Denmark).

### 2.2. Experimental Consumables and Reagents

PM_2.5_ Quartz Fiber Filters (PALL, Port Washington, NY, USA), n-Hexane, chromatography grade (Titan Scientific, Shanghai, China), Dichloromethane, chromatography grade (Titan Scientific, Shanghai, China), Acetonitrile, chromatography grade (Merck, Germany), Carbon Tetrachloride, chromatography grade (Adamas-beta, Shanghai, China), Tetrabromodiphenyl ether standard solution (Chemservice, Shanghai, China), Formic acid (Merck, Germany), PBS buffer (Kenon Biotechnology Co., Ltd., Weifang, China), 1.5 mL EP tube (Haige Biotechnology Co., Ltd., Fuzhou, China), 0.22 μm Hydrophilic Nylon Syringe Filter (Haige Biotechnology Co., Ltd.), 2 mL Brown Glass Vial (Haige Biotechnology Co., Ltd.), and Nitrogen gas (Xinzhongming Chemical Co., Ltd., Fuzhou, China).

### 2.3. Study Population and Sampling

#### 2.3.1. Epidemiological Survey

This study was conducted in accordance with the National Air Pollution (Haze) Health Impact Monitoring and Prevention Protocol. A total of 258 elderly residents were recruited from an urban–rural junction area (Taijiang District) in Fuzhou City for an on-site epidemiological investigation.

Inclusion Criteria: Permanent residency in the community for ≥5 years; age ≥ 60 years; regular daily routines, stable dietary habits, and fixed activity ranges; no acute respiratory or cardiovascular events within the past 3 months; consistent residence in the community during the past 3 months with no planned absences in the upcoming 3 months; good compliance with study procedures.

Exclusion Criteria: Individuals with cardiopulmonary diseases, cognitive impairment, or other severe conditions that could interfere with survey participation.

In this study, smoking was defined as consuming more than one cigarette per day within the past three months, and alcohol consumption as drinking more than once per week during the same period. Participants were categorized into cooking and non-cooking groups based on whether they had entered the kitchen to prepare meals during the study period. Fuel type was classified as gas fuel users versus non-gas fuel users. Air purifier use was divided into users and non-users. Indoor incense burning was categorized as present or absent. Educational attainment was grouped as post-secondary education (high school diploma or above) versus high school diploma or below. Additional covariates included age, sex, body mass index (BMI), daily time spent indoors, and indoor temperature and humidity.

#### 2.3.2. Individual PM_2.5_ Sampling

Based on preliminary findings indicating significant health effects from PM_2.5_ exposure on the third day of lagged exposure, and supported by pre-experimental results confirming that a 3-day sampling period meets detection limits, continuous 3-day monitoring was selected for environmental PM_2.5_ exposure assessment. In accordance with the Indoor Air Quality Standard (GB/T 18883-2022) [24], participants wore the Ultrasonic Personal Air Sampler V2 (UPAS V2) personal exposure particulate samplers for 72 h, with a flow rate of 1 L/min. Each sampling cycle consisted of 24 h of operation followed by a 60 s pause. Quartz filters specified in the national standard protocol were used for sample collection.

UPAS V2, developed by Access Sensor Technologies, is a wearable device designed to collect particulate matter from the user’s breathing zone onto a 37 mm filter cassette for subsequent laboratory analysis of mass concentration and chemical composition. Distinguished from conventional personal sampling pumps by its lightweight (230 g), near-silent ultrasonic pump, and integrated GPS, the UPAS V2 eliminates the need for cumbersome tubing and belt-mounted pumps. Its design allows for direct clipping onto the collar or shoulder strap, enabling continuous sampling for over 35 h during normal daily activities without causing significant noise disturbance.

In July–August 2023 and 2024, 129 community-dwelling older adults meeting the inclusion and exclusion criteria were enrolled in each sampling campaign, yielding a total of 258 unique participants. The samplers were worn near the chest at breathing-zone height during daytime activities and placed parallel to the bed during rest periods to simulate real-world exposure levels. The samplers were deployed among community-dwelling older adults, who were responsible for autonomously recharging the devices overnight while asleep to ensure uninterrupted 72 h continuous sampling. In the event of unexpected power failure or device shutdown during the sampling period, designated technical personnel were dispatched immediately to the participants’ residences within the community to perform on-site troubleshooting and maintenance. After sampling, filters were stored in transparent casings within low-temperature transport containers for laboratory analysis. PM_2.5_ mass concentration was calculated gravimetrically, and chemical compositions were quantified using GC/Q-TOF.

Daily visits by trained investigators documented participants’ time–activity patterns using standardized questionnaires. Instrument operation was verified regularly throughout the sampling period. After 3 days of PM_2.5_ monitoring, venous blood samples were collected by licensed physicians for subsequent metabolomic analysis.

### 2.4. Sample Analysis

Filters were pre- and post-weighed in a controlled environment (25 ± 1 °C, 50 ± 5% humidity) after 24 h equilibration. Two repeated measurements were taken for each filter, and the average value was used for concentration calculations. Field blanks and procedural controls were applied to minimize contamination during sampling and transport. PM_2.5_ concentration formula:

$$C=\frac{(W_{1}-W_{2})\times 10^{6}}{V_{0}}$$

C is the mass concentration (μg/m^3^), W_1_ and W_2_ are the masses of the filter membrane before and after sampling, respectively (g), and *V*_0_ is the sampling volume at standard conditions (m^3^).

### 2.5. GC/MS and LC/MS

PM_2.5_ samples were analyzed using an GC/Q-TOF. Separation was performed on an Agilent DB-5ms capillary column (30 m × 250 μm × 0.25 μm, Catalog No. 122-5532). Helium (99.999%) was used as the carrier gas at a constant flow rate of 1.3 mL/min. An amount of 2 μL of sample was injected in splitless mode using a multi-mode inlet (MMI). The inlet temperature was programmed from 70 °C (held for 0.2 min) to 325 °C at a rate of 600 °C/min (held for 7 min). The column oven temperature program was set as follows: initial temperature of 50 °C (held for 3 min), ramped to 320 °C at 6 °C/min (held for 7 min), with a total run time of 55 min and a 1 min equilibration time. Mass spectrometric detection was conducted in both Electron Ionization (EI) and Chemical Ionization (CI) modes. The transfer line and ion source temperatures were maintained at 280 °C and 300 °C, respectively. The quadrupole temperature was set to 175 °C. For EI mode, the ionization energy was 70 eV with an emission current of 35 μA. For CI mode, methane was used as the reagent gas (20% EPC), with an ionization energy of 115 eV and an emission current of 240 μA. Collision-induced dissociation (CID) was performed using nitrogen as the collision gas at a flow rate of 1.5 mL/min. Data were acquired in full scan mode with a mass range of *m*/*z* 55–700 and a scan rate of 5.00 spectra/s. Automatic tuning was performed prior to data acquisition.

Metabolites were separated on an Agilent SB-C18 column (2.1 × 50 mm, 1.8 μm) at 37 °C. The mobile phase comprised 0.1% formic acid in water (A) and acetonitrile (B) at a flow rate of 0.25 mL/min. The gradient profile was: 0–9 min, 2–60% B; 9–18 min, 60% B; 18–20 min, 60–100% B; 20–30 min, 100% B. The injection volume was 20 μL. MS detection was performed using an ESI source in both positive (5500 V) and negative (−4500 V) modes. The source temperature was 375 °C, with nitrogen as the collision gas. Data were acquired in EMS mode over a mass range of *m*/*z* 50–1500 at a scan rate of 4000 Da/s. The instrument was calibrated with tuning solution prior to analysis. Detailed information regarding the experimental protocols, instrumental parameters, and reagent specifications is provided in the Supplementary Methods.

### 2.6. LC-MS/GC-MS Profiling Data Conversion

GC-MS data were processed using Agilent MassHunter Qualitative Analysis (version 10.0) for peak picking, alignment, and matching. For LC-MS data, raw files were first converted to the .mzXML format via Agilent MassHunter utilities (including the LC-SQ ChemStation to MassHunter Translator and MSConvert), followed by peak picking, alignment, and matching on the XCMS Online platform (http://xcmsonline.scripps.edu).

Both GC-MS and LC-MS datasets were subjected to quality control filtering; air pollution and metabolites with median intensities below twice the noise threshold (signal-to-noise ratio, S/N < 2), or quantified in fewer than 30% of samples, were excluded. Samples with >50% missing values were removed; remaining missing values were imputed using the k-nearest neighbor (kNN) algorithm with 10 nearest neighbors. All metabolite data were subsequently normalized and log10-transformed to derive relative abundance values.

Ideally, the extracted ion chromatogram (EIC) of a valid feature ion should exhibit a narrow retention time span across samples, forming a compact peak cluster. However, due to matrix interference from plasma samples and instrument instability, retention time drifts frequently occurred in EICs of certain feature ions, introducing biases in response quantification and metabolite annotation. Thus, feature ions were further curated by manual inspection of their EIC profiles to exclude those with evident retention time shifts.

### 2.7. Exposure Characteristics Analysis

Raw GC-MS data were processed via automated annotation of chemical features using Agilent MassHunter Qualitative Analysis (version 10.0) with the integrated NIST20 mass spectral library. A minimum library match score of ≥70% was applied as the screening threshold for tentative annotation, not a criterion for definitive structural identification, consistent with the Schymanski et al. [25] and Xu et al. [26] confidence frameworks; under this system, all features passing the ≥70% match threshold were therefore assigned to CL2a (putative annotation), indicating high spectral similarity to a NIST20 reference entry without orthogonal validation via authentic standards. Detailed information on annotation confidence is provided in the Supplementary Methods. For each peak, the molecular formula linked to the highest-scoring library entry was retained. While non-targeted GC-MS offers robust analytical capability for low-polarity compounds [27], potential artifacts arising across the full sampling-to-analysis workflow must be considered when interpreting annotations [26,27]. Following software-based peak integration and manual curation of feature abundances, the assigned molecular formulas and quantitative results of all detected features were retained as tentative annotations. Expanded annotation details are provided in Table S2.

Descriptive analysis was performed on individual PM_2.5_ concentrations and their chemical components. For compound concentrations below the limit of detection (LOD), values were imputed as half of the lowest detected concentration. Spearman correlation analysis was employed to assess the relationships between individual PM_2.5_ (and its ester compounds) and meteorological/environmental factors. Given the large number of pairwise comparisons, the Benjamini–Hochberg false discovery rate (FDR) correction was applied to control for type I error inflation; correlations with an FDR-adjusted *p*-value < 0.05 were considered statistically significant. All statistical tests were two-sided, with a nominal significance level of α = 0.05. All analyses were conducted using R version 4.3.0.

### 2.8. Integrated Analysis of Relevant Metabolic Pathways

Metabolite identification was achieved by matching the accurate mass, isotopic patterns, and MS/MS spectra of detected metabolic features against public databases HMDB (www.hmdb.ca; accessed on 15 May 2025) and KEGG (www.genome.jp/kegg/; accessed on 15 May 2025). Matching criteria were set as follows: mass accuracy tolerance of 25 ppm and retention time (RT) tolerance of ±30 s. If a metabolic feature matched multiple MS/MS spectra, all matching spectra were used for identification. Metabolite identification required meeting three stringent criteria: narrow RT window, mass accuracy deviation < 10 ppm, and MS/MS spectral match based on ion comparison between experimental spectra and library entries.

Linear regression analyses were performed to identify metabolites significantly associated with PM_2.5_ and its chemical components. The continuous variables of log-transformed PM_2.5_ concentration and the concentrations of compounds with absolute correlation coefficients > 0.9 were included as independent variables. The relative abundance of identified metabolites served as the dependent variable, with age, sex, BMI, smoking status, and alcohol consumption included as covariates. The Benjamini–Hochberg false discovery rate (FDR) method was adopted for multiple comparison correction. Metabolites with an FDR-adjusted *p*-value < 0.05 were identified as significantly altered metabolites associated with PM_2.5_ exposure. The direction of association (positive or negative) was determined by the sign of the regression coefficient.

Metabolites significantly associated with exposure were imported into the MetaboAnalyst 5.0 online platform for metabolite set enrichment analysis (MSEA) integrated with KEGG pathways. MSEA aims to identify metabolic pathways that are significantly enriched within a set of metabolites through statistical analysis, thereby revealing their metabolic functions and mechanistic roles in biological systems. This analysis helps interpret the biological significance of metabolites, uncover associations between metabolites and diseases, drugs, or environmental factors, and explore potential biomarkers or therapeutic targets. The built-in Benjamini–Hochberg false discovery rate (FDR) correction in MetaboAnalyst 5.0 was applied to control for multiple pathway testing, and pathways with an FDR-adjusted *p*-value < 0.05 were considered statistically significant.

In MSEA, the FDR-adjusted *p*-value indicates whether the enrichment of a metabolite set in a specific pathway is statistically significant (i.e., unlikely due to random chance). Generally, a smaller *p*-value indicates a higher degree of enrichment and greater pathway significance. The enrichment factor (EF) is a metric that reflects the ratio of the proportion of metabolites in a given pathway within the metabolite set of interest to the proportion in the background set. A larger EF signifies a better enrichment effect and greater importance of the pathway. The EF is calculated as follows:

Enrichment Factor (EF) = (*n*/*N*)(*m*/*M*)

*m*: number of metabolites in the metabolite set of interest belonging to the specific pathway

*M*: number of background metabolites belonging to the specific pathway

*n*: total number of metabolites in the metabolite set of interest

*N*: total number of background metabolites

### 2.9. Quality Control

Rigorous quality control (QC) procedures were implemented throughout all stages of the field survey, including site selection, study design, investigator training, field sampling, laboratory analysis, and data management, to ensure the reliability and validity of the results.

#### 2.9.1. Field Quality Control

The study sites were selected based on the National Air Pollution Monitoring Program protocol. Selected communities exhibited high resident compliance and were located in proximity to national ambient PM_2.5_ monitoring stations. The questionnaire was developed and revised by the author based on the Protocol for Urban Indoor Health Effects Survey and Evaluation of Protective Measures issued by the Chinese Center for Disease Control and Prevention, as well as existing validated questionnaires. All investigators underwent unified training and participated in a pre-survey to standardize operational methods.

Prior to sampling, all samplers were calibrated to minimize measurement bias. A two-week validation test was conducted to assess inter-instrument agreement across three types of monitors, revealing good consistency with an average error of less than 10%. During the fieldwork, investigators assisted participants in correctly wearing individual samplers. Flow rates of all sampling pumps were recalibrated uniformly during filter changes to maintain constant sampling flow. Throughout the sampling period, investigators conducted twice-daily household visits (morning and afternoon) to verify pump functionality and the positioning of sampling heads, ensuring samples accurately reflected participants’ particulate matter exposure. For each batch of exposure measurements, at least one duplicate sample and one field blank were included. Samples with daily operation times of less than 20 h or flow rate drops exceeding 10% during sampling were excluded from analysis.

#### 2.9.2. PM_2.5_ Sampling Quality Control

• Field Blanks: Sealed blank filter membranes were taken to the field site, installed and removed following the standard sampling procedure without drawing air, and then transported back to the laboratory for processing alongside actual samples.
• Pre-sampling Preparation: Glass fiber filters were wrapped in aluminum foil (with openings) and baked in a muffle furnace at 400 °C for 5 h to remove organic matter and enhance membrane toughness. Filters were conditioned in a constant temperature and humidity chamber (25 ± 1 °C; 50 ± 5% RH) for at least 24 h prior to weighing.
• Sampling Procedures: Filter integrity was inspected before installation; membranes were required to be smooth, uniform, contaminant-free, and free of pinholes or creases. Non-metallic, non-serrated tweezers were used for handling. Before each sampling event, the sampler’s ambient temperature (error ≤ ±2 °C) and barometric pressure (error ≤ ±1 kPa) sensors were calibrated.
• Sample Transport and Storage: Post-sampling, filters were placed in sealed cases, transported in light-proof bags, and stored at 4 °C. Samples were weighed within 30 days of collection, with chemical extraction and analysis conducted promptly thereafter.
• Weighing Procedures: Analytical balances were calibrated before each weighing session. The temperature and humidity in the balance room were maintained consistent with filter conditioning conditions (25 ± 1 °C; 50 ± 5% RH).

#### 2.9.3. Laboratory Procedure Quality Control

All instruments were calibrated prior to analysis. High-purity reagents were utilized for standard solution preparation and sample processing. Calibration curves were established for each element, with mandatory linearity correlation coefficients (R^2^) ≥ 0.999. Each analytical batch included one laboratory reagent blank, one field blank, and one quality control sample. Standard operating procedures were strictly adhered to during sample preparation and analysis.

#### 2.9.4. Data Quality Control

Ambient PM_2.5_ data were audited according to the National Work Plan for Monitoring and Protecting Human Health from Air Pollution (Haze). Values below the limit of detection (LOD) were substituted with half of the LOD value. Logical checks were performed on all questionnaire variables. Double data entry and consistency checks were conducted using EpiData 3.1 software to ensure data authenticity, accuracy, and reliability.

## 3. Results

### 3.1. General Characteristics of Study Participants

After screening based on inclusion and exclusion criteria, a total of 258 elderly community participants were enrolled in this study. Among them, 114 were male and 144 were female, with a mean age of 71.37 ± 7.07 years and an average BMI of 24.11 ± 3.01. Participants spent an average of 20.98 h indoors and 3.01 h outdoors daily. Over half of the participants engaged in physical activity for more than 0.5 h per day. Educational levels were distributed as follows: 76 participants had elementary school education or below, 66 had junior high school education, 60 had high school education, and 56 had college education or above. Smoking and drinking were reported by 31 and 44 participants, respectively, while 64 participants were exposed to passive smoking, with 32 exposed for at least 0.5 h daily (Table 1).

### 3.2. Exposure Characteristics of PM_2.5_ and Its Putatively Annotated Chemical Components

A total of 258 individual PM_2.5_ samples were collected, with an average environmental temperature of 29.57 °C and relative humidity of 74.76%. The mean concentration of individual PM2.5 was 38.06 μg/m^3^, ranging from 2.684 to 268.947 μg/m^3^. Based on untargeted GC-MS, a total of 3071 compounds exhibiting ester-like features were extensively screened from PM_2.5_ sampling filters. Ester compounds were the predominant chemical components, with cholesteryl methyl carbonate showing the highest average concentration of 50.37 ng/m^3^ (Table 2).

**Figure 1:**
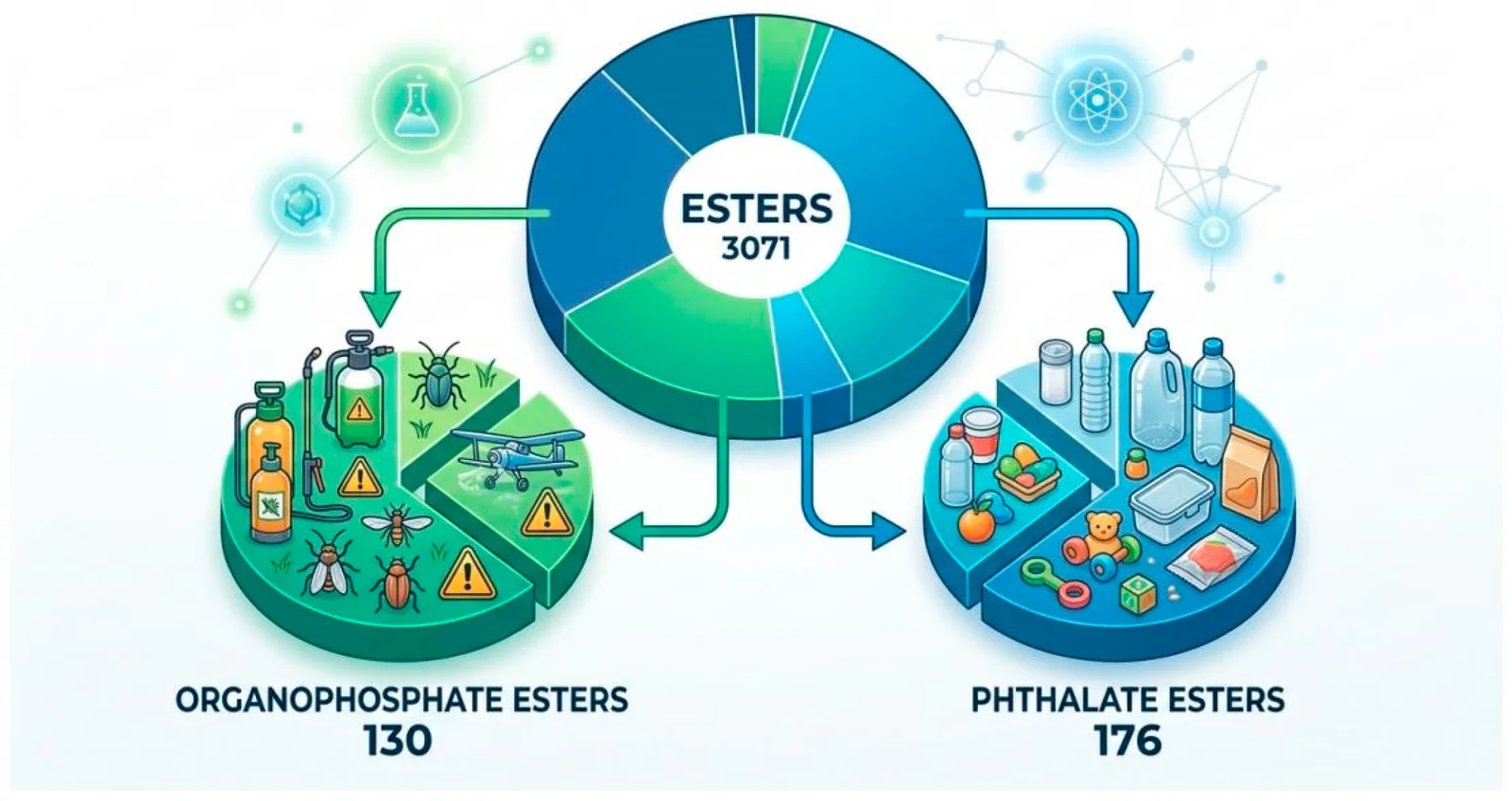
Classification and Major Subgroups of Screened Putatively Annotated Ester Compounds.

Within the extensive screening of 3071 unique ester-like features, a targeted analytical focus was applied based on the density of existing exposure and health effects research. The data segregation revealed that two specific subclasses constituted the primary subjects of prior epidemiological and toxicological studies: organophosphate esters (OPEs, *n* = 130) and phthalate esters (PAEs, *n* = 176) (Figure 1).

Our initial screening putatively annotated 2765 ester compounds distinct from the well-characterized phthalate and organophosphate ester subclasses. From this extensive ester pool, a refined subset of 20 compounds was selected for in-depth investigation. The primary selection criterion was that their mean concentrations detected in preliminary environmental and biological samples exceeded the maximum concentrations reported for common OPEs and PAEs contaminants; quantitative levels and putative annotations of OPEs and PAEs are detailed in Tables S1 and S2.

This concentration-based prioritization highlights these 20 esters as potential high-exposure agents requiring immediate scrutiny. The selected compounds will be subjected to a comprehensive analysis integrating key environmental factors. This integrated assessment aims to elucidate the sources and drivers of their elevated environmental presence.

Furthermore, this prioritized subset of 20 high-concentration, non-target esters has been designated for inclusion in a subsequent metabolomics study. This next-phase analysis will specifically investigate their potential biological effects and mode of action by profiling the endogenous metabolic alterations associated with their exposure.

**Figure 2:**
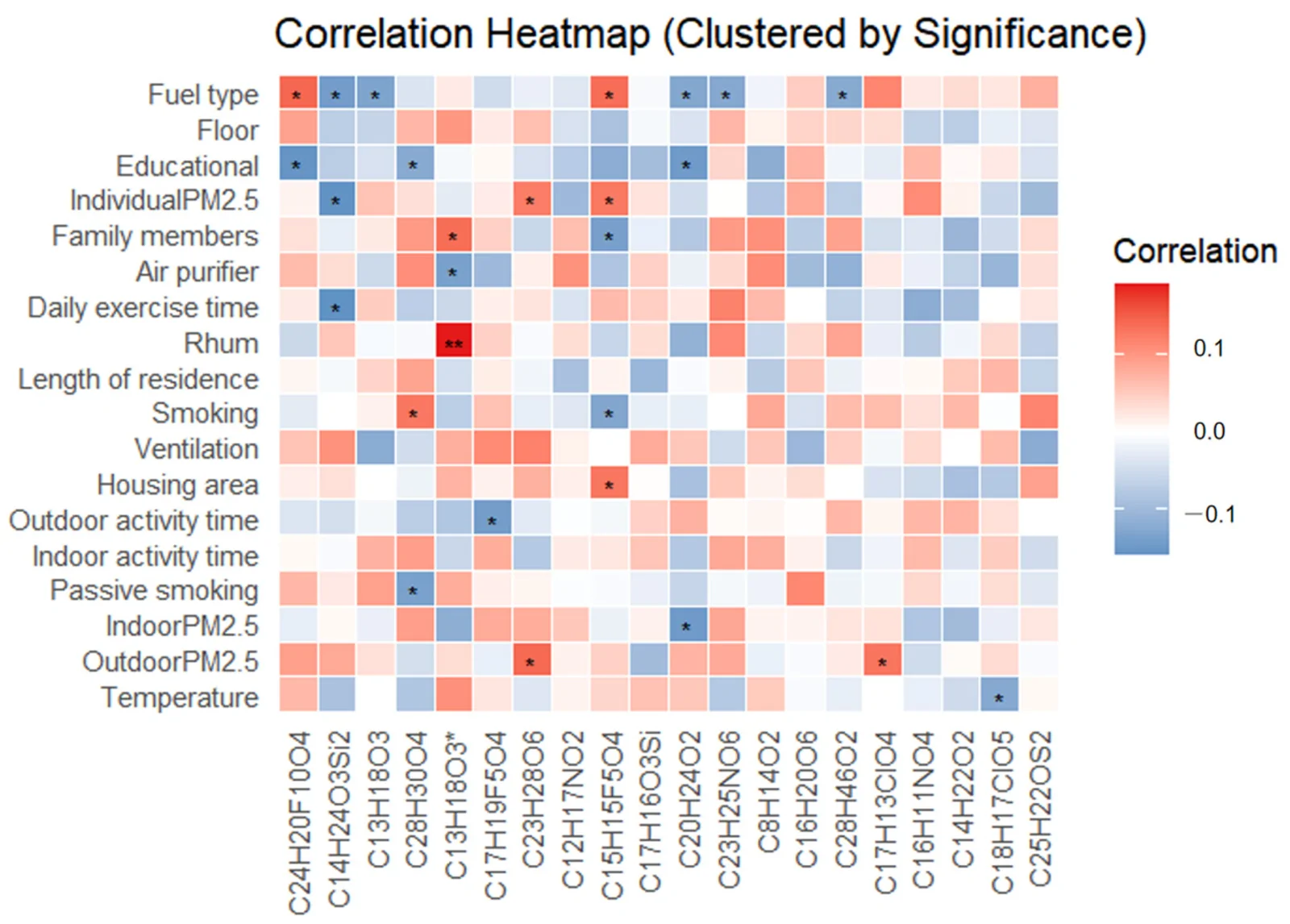
Heatmap of Regression Analysis Results for Individual Ester Exposure and Influencing Factors. The color in the heatmap represents the standardized regression coefficients. * indicates statistical significance, with * *p* < 0.05, ** *p* < 0.01.

Spearman correlation analysis revealed significant associations between ester compounds and environmental factors (e.g., temperature, humidity), residential characteristics (e.g., housing area, household size), and behavioral factors (e.g., smoking, cooking). Regression analysis further demonstrated that ester compound exposure was influenced by fuel type, educational level, and indoor activities (Figure 2).

### 3.3. Metabolic Enrichment of PM_2.5_-Borne Ester Compounds

**Figure 3:**
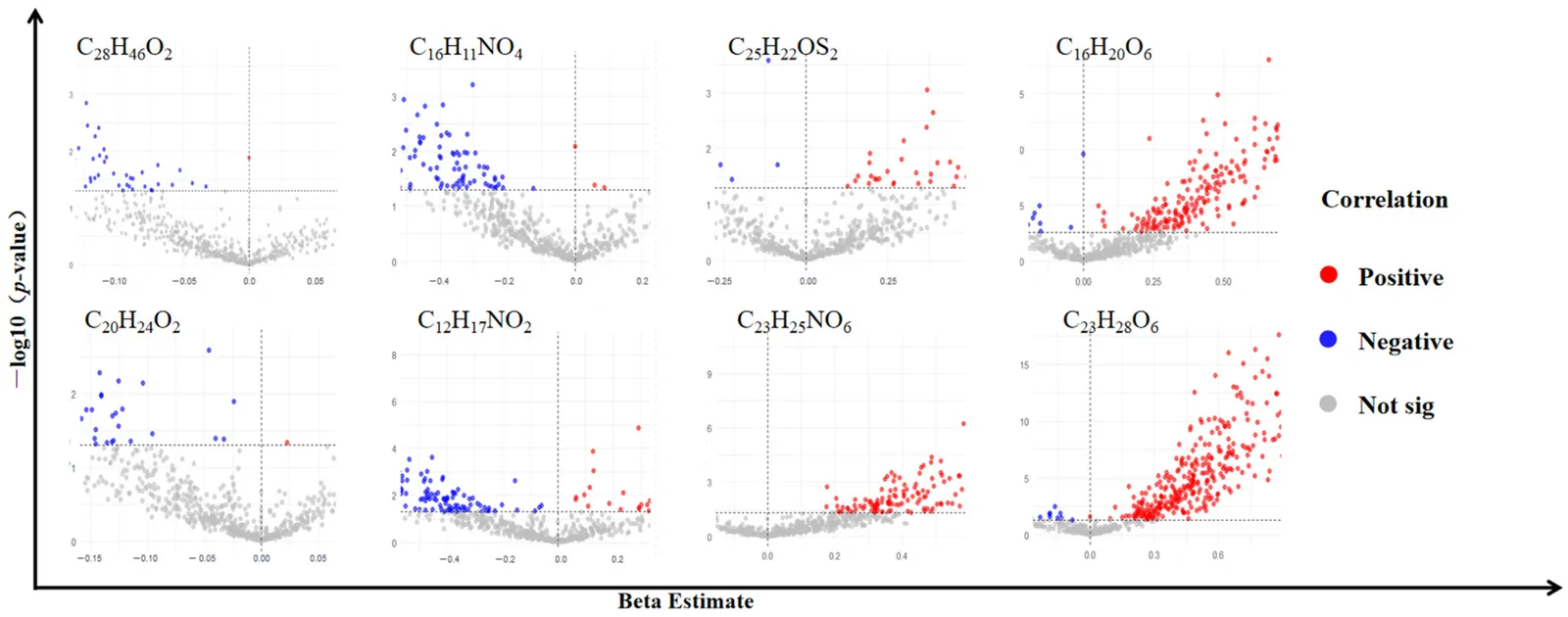
Volcano Plot of Plasma Metabolites Associated with Ester Exposure in PM_2.5_.

LC-MS analysis of 258 plasma samples identified 609 feature ions from an initial pool of 104,603 peaks. After database matching, 494 metabolites were confirmed (387 in positive ion mode, 107 in negative ion mode). Linear regression models, adjusted for covariates (e.g., age, gender, BMI), identified significant associations between PM_2.5_-borne ester compounds and metabolite abundance (*p* < 0.05). Volcano plots visualized differentially expressed metabolites, with red dots indicating positive correlations and blue dots indicating negative correlations (Figure 3). The other 12 volcano plots can be found in Supplementary Figures S1 and S2.

Metabolic pathway enrichment analysis using MetaboAnalyst5.0 revealed that PM_2.5_ exposure significantly affected pathways such as thiamine metabolism, linoleic acid metabolism, glycerophospholipid metabolism, and steroid hormone biosynthesis (Figure 3).

**Figure 4:**
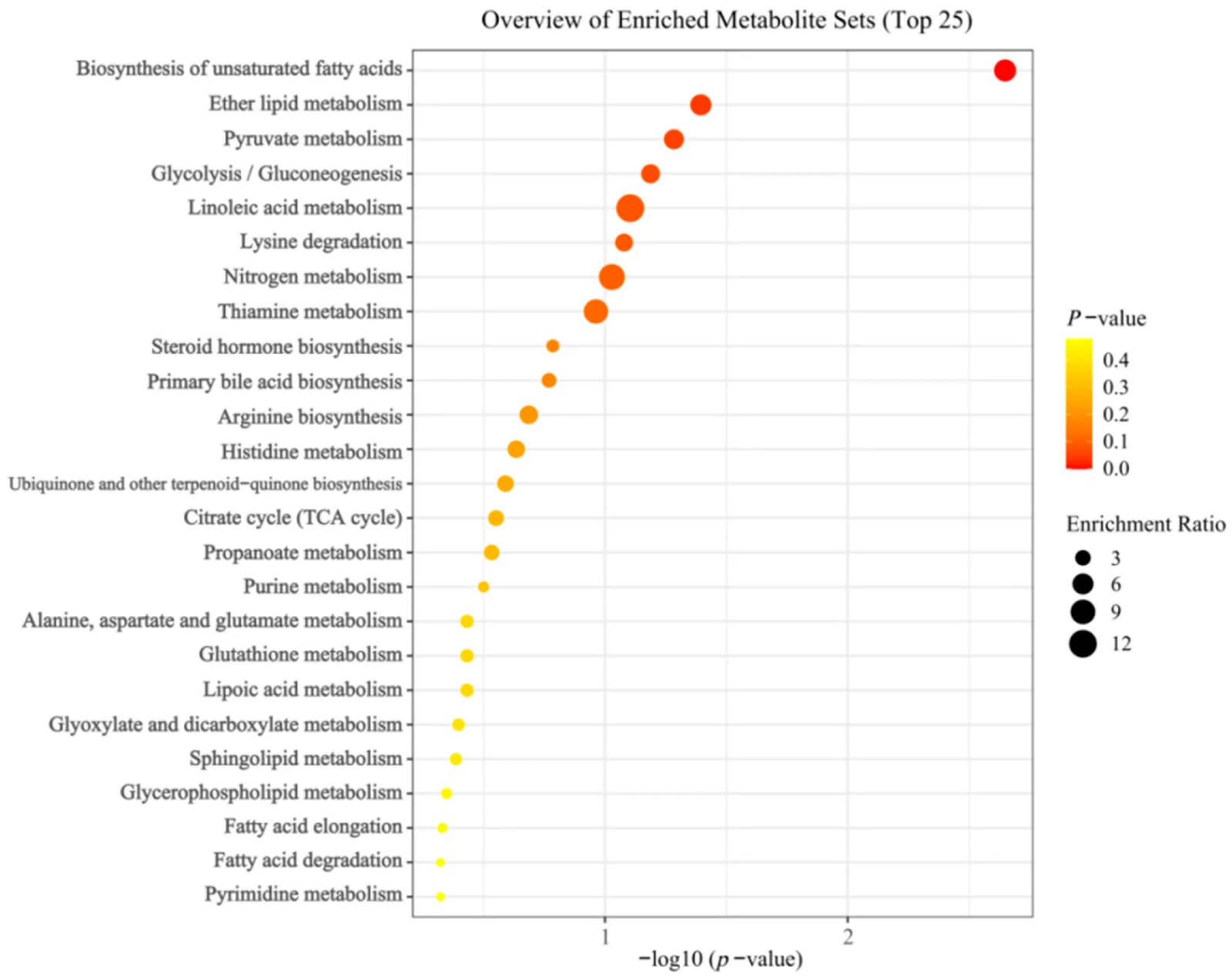
Metabolic Pathway Enrichment Results for PM_2.5_.

Analysis of the main metabolic pathways enriched for plasma metabolites influenced by PM_2.5_-bound putatively annotated ester compounds reveals that the affected pathways encompass numerous fundamental biochemical processes within organisms (Figure 4). These include linolenic acid and linoleic acid metabolism, acetyl group transfer to mitochondria, glycolysis, steroid hormone biosynthesis, glutamate metabolism, branched-chain fatty acid oxidation, peroxisomal phytanic acid oxidation, the urea cycle, pyrimidine metabolism, the Warburg effect, and others.

Integrating the findings from Figure 3, and to gain deeper insight into the mechanisms by which ester compounds influence human metabolic pathways, five specific esters with a higher number of associated differential plasma metabolites were selected for further metabolic pathway enrichment analysis. These putatively annotated compounds are C_23_H_28_O_6_, C_16_H_20_O_6_, C_16_H_11_NO_4_, C_12_H_17_NO_2_, and C_23_H_25_NO_6_. The remaining results are illustrated in Supplementary Figures S3–S7.

Further investigation into the metabolic pathways associated with exposure to C_23_H_28_O_6_ (putatively annotated as cholesteryl formate), a major ester compound in PM_2.5_ with numerous differential metabolites, shows that the impacted pathways cover many basic biological processes. These include steroid biosynthesis, fatty acid biosynthesis, glutamate metabolism, etc., suggesting that this compound may influence physiological processes such as lipid metabolism and hormone synthesis through these pathways.

The metabolic pathways affected by C_16_H_20_O_6_ (putatively annotated as benzylmalonate diester) encompass fatty acid biosynthesis, methylhistidine metabolism, steroid biosynthesis, glycine and serine metabolism, pyruvate degradation, bile acid biosynthesis, and arachidonic acid metabolism. This involves various physiological and biochemical processes such as lipid metabolism, energy metabolism, and neurotransmitter regulation.

Enrichment results for C_16_H_11_NO_4_ (putatively annotated as 1-phenanthrenecarboxylicacid, 10-nitro-, methyl ester) indicate effects on physiological and biochemical processes including pyrimidine metabolism, phenylacetate metabolism, carnitine synthesis, branched-chain fatty acid oxidation, peroxisomal phytanic acid oxidation, the urea cycle, and ammonia recycling.

C_12_H_17_NO_2_ (putatively annotated as 4-Picolinic acid methyl ester) is primarily involved in pathways such as fatty acid biosynthesis, pyrimidine metabolism, β-oxidation of very long-chain fatty acids, and purine metabolism.

C_23_H_25_NO_6_ (putatively annotated as N-benzoyl-N-methylamino malonic acid dimethyl ester) affects pathways including purine metabolism, nicotinate and nicotinamide metabolism, glutamate metabolism, pyrimidine metabolism, and phenylacetate metabolism, potentially interfering with the body’s biological signal transduction pathways.

**Figure 5:**
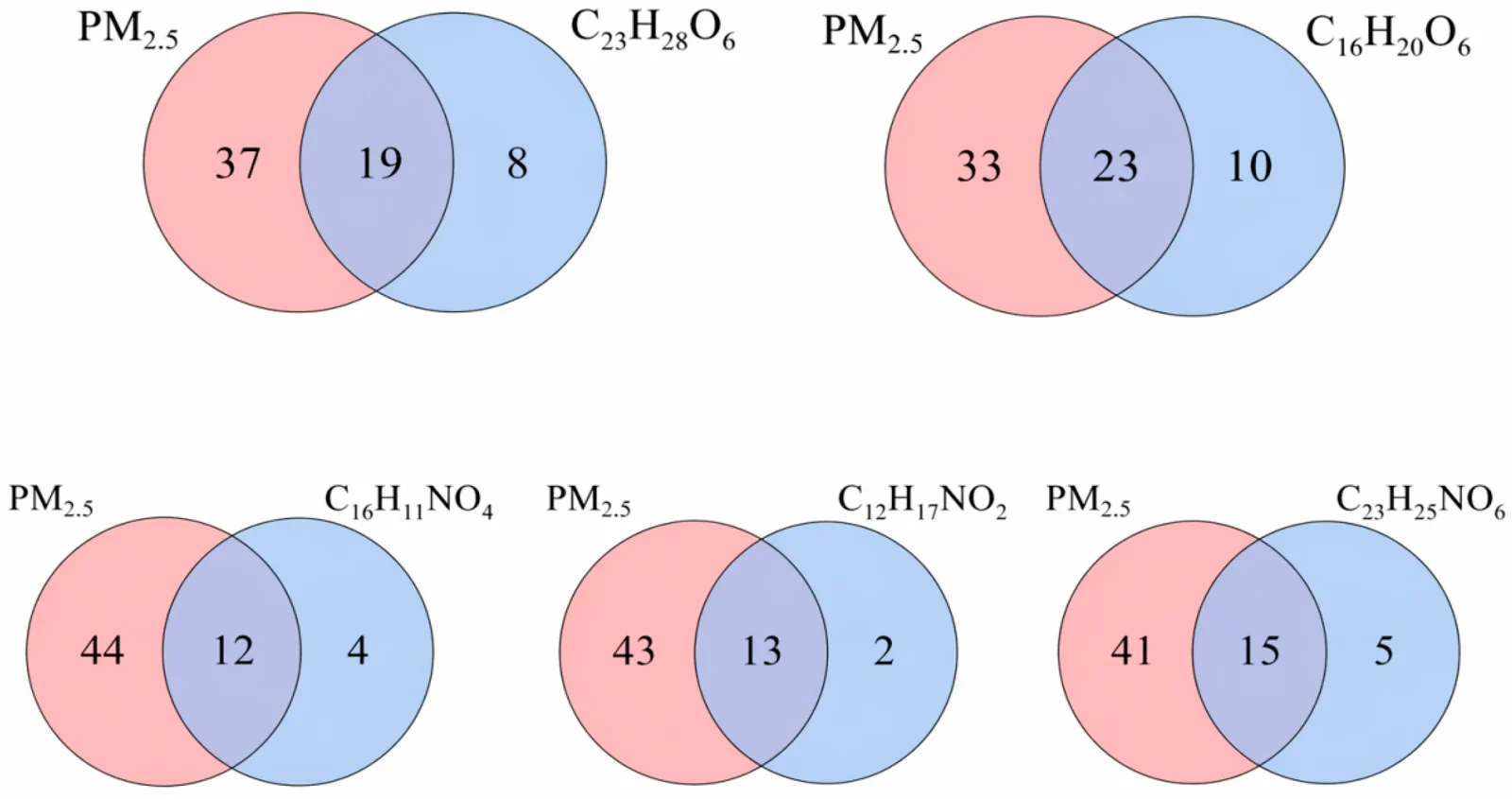
Venn Diagrams of Enriched Metabolic Pathways for PM_2.5_ and Five Putatively Annotated Ester Compounds.

Following the enrichment of target esters, we integrated the differential metabolites indicated by these compounds for subsequent enrichment and pathway analyses. We integrated the analysis of the aforementioned ester compounds and retained only the overlapping/co-occurring differential metabolites identified in the PM_2_._5_ analysis (Figure 5). The results demonstrated that PM_2.5_-bound esters exhibited significant associations with biological processes related to steroid metabolism, as well as glycolysis and gluconeogenesis.

**Figure 6:**
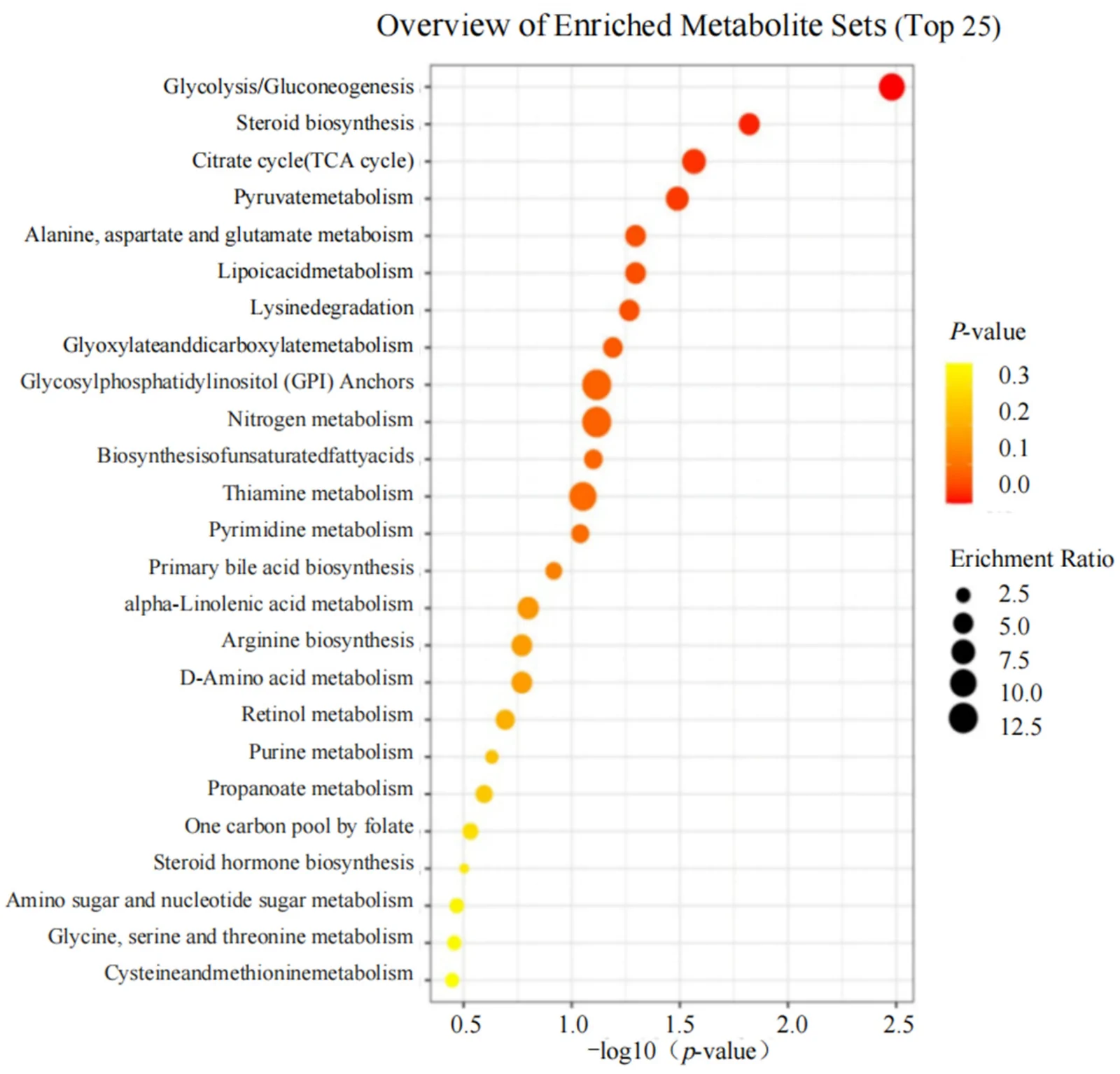
Metabolic Pathway Enrichment Results for Main Putatively Annotated Ester Compounds.

Pathway enrichment analysis revealed that these co-existing metabolites were predominantly mapped to five core pathways: Steroid biosynthesis, Glycolysis/Gluconeogenesis, Glycerophospholipid metabolism, and Purine/Pyrimidine metabolism. Topological visualization of these pathways highlighted distinct patterns of alterations. In Steroid biosynthesis, key intermediates such as Squalene and Cholesterol esters (e.g., CE(16:1)) exhibited significant upregulation, coupled with alterations in Vitamin D_3_ metabolites. This suggests that PM_2.5_-bound esters may modulate the late-stage modification and esterification processes critical for maintaining steroidal homeostasis (Figure 6).

**Figure 7:**
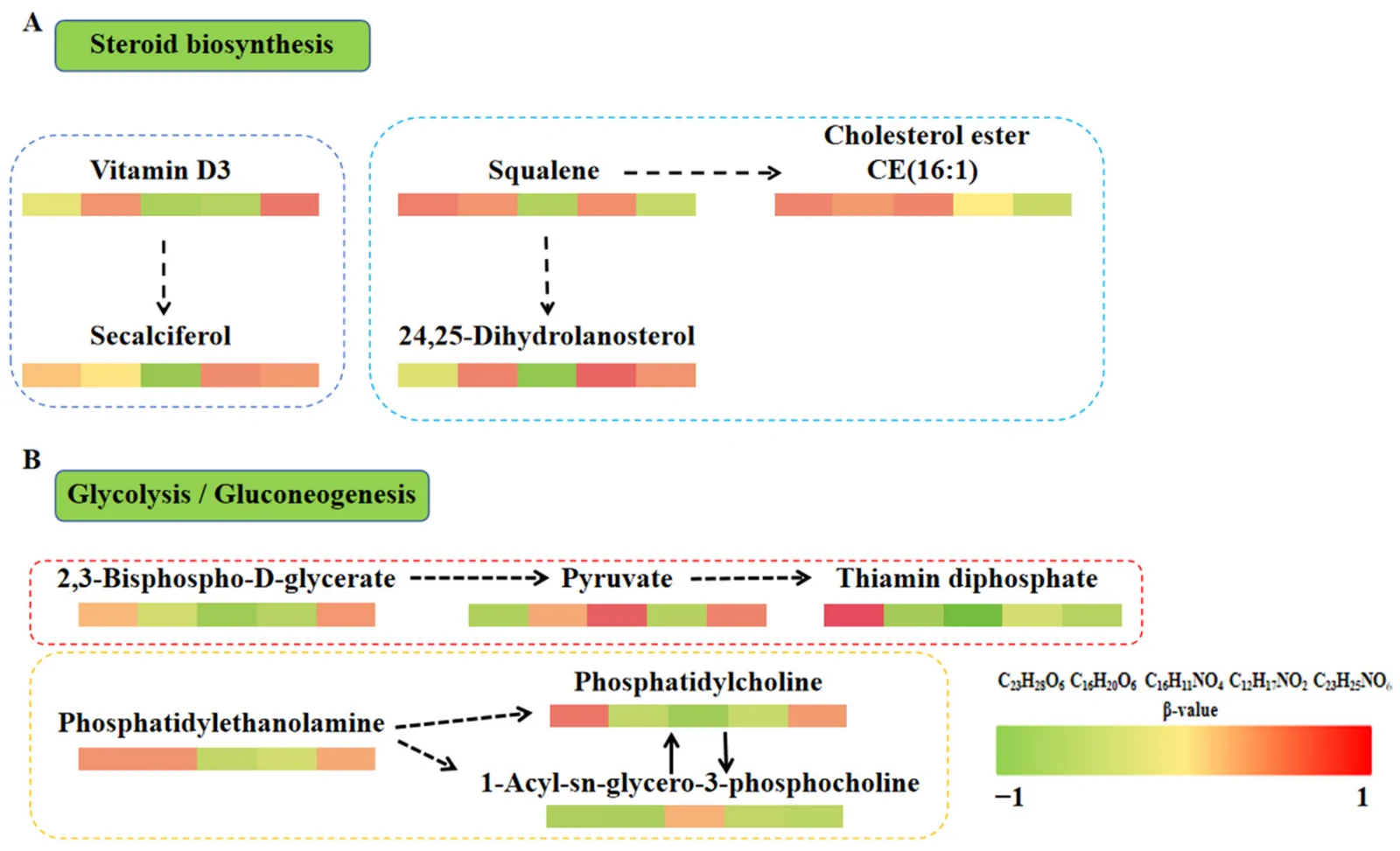
PM_2.5_-Bound Ester-Associated Alterations in Steroid and Glycolysis/Gluconeogenesis Pathways. (**A**) Metabolic features significantly altered in the Steroid biosynthesis pathway. Dashed arrows indicate indirect biotransformation processes. Key metabolites include Vitamin D3, Secalciferol, Squalene, 24,25-Dihydrolanosterol, and Cholesterol ester (CE(16:1)). (**B**) Metabolic features significantly altered in the Glycolysis/Gluconeogenesis and related phospholipid pathways. Dashed arrows denote indirect biotransformation. Key metabolites include 2,3-Bisphospho-D-glycerate, Pyruvate, Thiamin diphosphate, Phosphatidylethanolamine, 1-Acyl-sn-glycero-3-phosphocholine, and Phosphatidylcholine. Note: The color scale represents the β-value, ranging from −1 (green, downregulated) to 1 (red, upregulated).

PM_2.5_ and its ester components are associated with oxidative stress and inflammatory responses by modulating lipid metabolism pathways such as the Steroid Biosynthesis Pathway. Key intermediate metabolites in this pathway exhibited significant changes. Both Squalene and 24,25-Dihydrolanosterol showed a significant downward trend, suggesting that early steps of steroid synthesis were inhibited. Additionally, the end product Cholesterol ester CE(16:1) was also significantly reduced. Notably, the conversion of Vitamin D_3_ to Secalciferol displayed positive regulation, indicating the presence of a salvage synthesis mechanism under specific conditions. Regarding glucose metabolism, metabolite fluctuations were relatively drastic. Both Pyruvate and Thiamin diphosphate showed significant negative regulation, implying that the downstream metabolism of pyruvate and the activation of Vitamin B1 were hindered. Furthermore, the levels of the lipid-related intermediate 1-Acylsn-glycero-3-phosphocholine decreased, while the downstream products Phosphatidylethanolamine and Phosphatidylcholine exhibited an upward trend. This reflects the remodeling of cell membrane phospholipid composition and a possible compensatory synthetic response (Figure 7).

## 4. Discussion

### 4.1. Analysis of Exposure Characteristics

This study analyzed the personal PM_2.5_ exposure characteristics of elderly residents in a community located in a suburban–rural fringe area of Fuzhou City. The findings revealed that the average personal PM_2.5_ concentration was 38.06 μg/m^3^, with ester compounds being the predominant chemical components. Among these, C_28_H_46_O_2_ (putatively annotated as cholesteryl formate) exhibited the highest concentration. The elevated level of cholesteryl formate may be associated with its role as a degradation product of certain organic compounds, suggesting that the PM_2.5_ to which individuals are exposed may contain complex chemical components from various indoor and outdoor pollution sources. The correlation heatmap indicated a close association between ester compounds and combustion types, implying that they may originate simultaneously from combustion processes or the evaporation of chemicals. Furthermore, individual exposure levels to ester compounds were significantly influenced by multiple factors, including meteorological conditions, residential characteristics, and behavioral activities. The significant differences in sources among different categories of compounds highlight the need for targeted improvements in the residential environment and behavioral interventions to reduce exposure risks.

Lower floors typically have poorer ventilation, which may lead to the accumulation of ester compounds. Research indicates that phosphate esters primarily adsorb onto PM_2.5_. Exposure to phosphate compounds usually occurs through the ingestion or inhalation of larger particles; exposure mediated by fine particulate matter may lead to exceedances of individual health limits [28]. Concentrations increase during hot seasons, suggesting that temperature is also an important factor influencing personal exposure to esters [29]. Phthalate esters (also known as plasticizers) primarily originate from the indoor use of products containing plasticizers or emissions from plastic factories and various other fuel sources [30,31]. Indoor incense burning, such as during aromatherapy, releases a variety of organic compounds. According to a study in Baoding City, coal combustion pollution and traffic pollution may also increase the concentration of ester substances in PM_2.5_. Large furniture, especially newly purchased items, may contain volatile organic compounds as part of the materials or coatings, leading to increased concentrations of certain compounds. Some ester compounds, such as Trifluralin, may originate from antibacterial agents added to air humidifiers or sofa foam materials. Increased humidity facilitates their release, while the use of humidifiers and prolonged sitting on sofas increase exposure opportunities. Diethyl oxalate may adsorb to dust, and frequent sweeping reduces its residual levels in the indoor environment. These complex associations indicate that individual exposure levels are influenced by a combination of meteorological conditions, residential physical conditions, and behavioral habits [32].

### 4.2. Metabolomics Analysis

We distinguish below between empirically observed metabolic associations and putative mechanistic interpretations extending beyond our primary data.

Integrated analysis of the metabolic pathways associated with PM_2.5_ and its carried putatively annotated ester compounds showed that the commonly affected pathways primarily involve multiple biological processes, including lipid metabolism, hormone and bile acid synthesis, amino acid metabolism, glucose/lipid metabolism, and signal transduction. Our observation that PM_2.5_ esters suppress key intermediates in steroid biosynthesis (e.g., squalene, lanosterol) while altering cholesterol ester profiles aligns with and extends recent toxicological evidence. While Zhang et al. linked PM_2.5_ components to metabolic syndrome in older adults [6], our study pinpoints specific ester structures as potential drivers of this association. The downregulation of squalene observed here is consistent with the inhibitory effects of certain industrial esters on lanosterol synthase activity reported in vitro [7]. As a putative mechanistic explanation, this alteration likely stems from the “ester bond reactivity” motif discussed in our introduction: esterase-mediated biotransformation of these compounds may compete for or deplete cellular pools of carboxylesterases and cytochrome P450 enzymes, thereby diverting resources away from endogenous cholesterol and steroid synthesis [8]. Furthermore, the concurrent dysregulation of glycolysis/gluconeogenesis (e.g., suppression of pyruvate and thiamine diphosphate) and glycerophospholipid metabolism represents a putative link to the epidemiological observations of Sun et al., who reported increased heart rate and metabolic disturbances in middle-aged populations exposed to the PM_2.5_ chemical exposome [2]. The remodeling of phospholipids (decreased lysophosphatidylcholines, increased phosphatidylethanolamines) suggests that these esters may compromise pulmonary and systemic endothelial barrier integrity, triggering a low-grade inflammatory state that exacerbates insulin resistance [20]. These mechanistic inferences, however, remain speculative and require targeted experimental validation.

Among other enriched pathways are glycerophospholipid metabolism, unsaturated fatty acid biosynthesis, and arachidonic acid metabolism. Based on the prior literature linking these pathways to cardiometabolic risk [33], we hypothesize that such perturbations may contribute to fatty acid metabolism imbalances, insulin resistance, and atherosclerotic processes, which could elevate the risk of cardiovascular disease. Associations between putatively annotated PM_2.5_ and alterations in steroid synthesis may plausibly contribute to hormonal imbalances, potentially leading to increased blood pressure, changes in heart rate, and atherosclerosis, thus elevating the risk of heart disease and hypertension [34]. Observed alterations in bile acid metabolism raise the possibility of impaired hepatic function, which could increase the risk of liver pathologies including steatotic liver disease and cirrhosis [35]. During amino acid metabolism, by inhibiting the activity of lysine degradation enzymes, PM_2.5_ may modulate neurotransmitter metabolism and immune regulation, increasing the risks of neurodegenerative diseases, diabetes, and metabolic syndrome [36]. In signal transduction processes, by inhibiting key molecules in purine metabolism and coinciding with alterations in cell communication mediated by GPI-anchored proteins, PM_2.5_ may modulate pathways like MAPK/PI3K-Akt, potentially exacerbating chronic inflammation and immune imbalance and promoting the development of autoimmune diseases and metabolic disorders [37]. We emphasize that these clinical implications are hypothetical extensions of our metabolic findings rather than demonstrated outcomes.

### 4.3. Limitations and Future Research Directions

Several methodological constraints warrant consideration regarding the identification of ester compounds. While GC-MS coupled with NIST library matching provided a broad characterization of PM_2.5_ constituents, the identification of numerous compounds remains at the tentative annotation level. Notably, a significant proportion of detected features lacked plausible environmental precedence as known pollutants. These anomalies are hypothesized to stem from in-source thermal degradation (e.g., peroxy-oxidation artifacts under high inlet temperatures) or erroneous spectral library assignments rather than genuine atmospheric occurrence [26]. Critically, while the aggregated concentrations of these unidentified ester fractions often exceeded those of established pollutants like OPEs and PAEs, definitive structural elucidation is lacking. Consequently, these compounds necessitate validation via authentic standards and orthogonal techniques in future studies to confirm their chemical identities and environmental relevance [38].

Furthermore, caution is advised regarding the generalizability of our epidemiological findings. This study focused exclusively on relatively healthy, community-dwelling older adults without pre-existing cardiorespiratory comorbidities, limiting the extrapolation of our results to clinical populations. However, the characterization of potential confounders remains incomplete. Occupational exposure histories were not sufficiently detailed, precluding adjustment for lifetime dust exposure common among individuals with manual labor backgrounds. Similarly, smoking data lacked granularity regarding passive smoke exposure, quit histories, and pack–years, while the prevalence of respiratory symptoms was not documented [39]. The absence of data on comorbid conditions, polypharmacy, and their metabolic implications further complicates the isolation of PM_2.5_-specific effects in this elderly cohort. Additionally, the single-season (summer) sampling design restricts inferences about seasonal variations in PM_2.5_ chemistry and toxicity [40,41]. Residual confounding from crudely categorized environmental factors, indoor furnishing off-gassing, and micro-environmental exposures may persist despite adjustments [42]. Future research should implement multi-season, multi-center cohorts that integrate refined exposure assessments, comprehensive occupational and clinical covariate data, and detailed lifestyle questionnaires to disentangle these complex interactions.

## 5. Conclusions

This study integrates personal exposure monitoring with untargeted metabolomics to identify potential associations between under-characterized PM_2.5_-associated ester constituents and observed perturbations in biological processes related to systemic metabolic homeostasis among community-dwelling older adults. Our findings demonstrate that the health burden of PM_2.5_ is critically mediated by its chemical complexity rather than mass concentration alone, specifically through the suppression of steroid biosynthesis (e.g., squalene, lanosterol) and the remodeling of glycerophospholipid metabolism. These metabolic alterations are consistent with prior epidemiological observations of associations between PM_2.5_ exposure and insulin resistance and cardiovascular risk and represent candidate pathways that may contribute to the heightened susceptibility of older adults to air pollution-related health effects—a hypothesis requiring experimental validation, underscoring the exploratory and hypothesis-generating nature of this work. We propose that these ester constituents act as xenobiotic mimics, competing for esterase resources and thereby interfering with endogenous lipid processing and hormonal regulation—a risk exacerbated by age-related immunosenescence and altered detoxification capacities. Furthermore, the strong associations observed with indoor activities and housing characteristics underscore the need to shift from mass-based metrics toward chemical-specific risk assessments in environmental health policy. Future research should prioritize longitudinal designs to establish causal links between these specific ester exposures and clinical endpoints, alongside toxicological validation to examine their direct perturbation of metabolic pathways.

## Figures and Tables

**Table 1:** Basic Characteristics of the Study Subjects.

Characteristics	Mean ± SD/N (%)
Age (years)	71.37 ± 7.07
BMI (kg/m^2^)	24.11 ± 3.01
Indoor activity time (h/day)	20.98 ± 2.71
Outdoor activity time (h/day)	3.01 ± 2.71
Gender	
Male	114 (44.19)
Female	144 (55.81)
Education level	
Primary school and below	28 (10.85)
Primary school	48 (18.60)
Junior high school	66 (25.58)
High school	60 (23.26)
University and above	56 (21.71)
Smoking	
No	227 (87.98)
Yes	31 (12.02)
Second-hand smoke	
No	194 (75.19)
Yes	64 (24.81)
Exposure to second-hand smoke	
<0.5 h/day	226 (87.60)
0.5–1 h/day	16 (6.20)
>1 h/day	16 (6.20)
Alcohol drinking	
No	214 (82.95)
Yes	44 (17.05)

**Table 2:** Basic Characteristics of Individual PM_2.5_ and Its Putatively Annotated Ester Components.

Component	N	Mean ± SD	Minimum	Maximum
T (°C)	258	29.57 ± 2.28	15.370	34.500
Rhum (%)	258	74.76 ± 12.03	31.681	99.000
Individual PM_2.5_ (μg/m^3^)	258	38.06 ± 48.38	2.684	268.947
C_28_H_46_O_2_ (ng/m^3^)	258	50.37 ± 444.62	1.827	7118.377
C_13_H_18_O_3_ (ng/m^3^)	258	42.76 ± 396.85	0.712	6353.121
C_23_H_28_O_6_ (ng/m^3^)	258	33.94 ± 49.69	2.361	210.985
C_16_H_20_O_6_ (ng/m^3^)	258	31.60 ± 40.18	3.692	198.127
C_12_H_17_NO_2_ (ng/m^3^)	258	31.06 ± 27.43	0.106	82.069
C_17_H_16_O_3_Si (ng/m^3^)	258	30.81 ± 252.64	1.341	3952.810
C_13_H_18_O_3_ * (ng/m^3^)	258	30.49 ± 31.30	0.195	219.278
C_18_H_17_ClO_5_ (ng/m^3^)	258	22.17 ± 15.23	7.880	47.541
C_25_H_22_OS_2_ (ng/m^3^)	258	21.16 ± 14.56	5.992	67.881
C_8_H_14_O_2_ (ng/m^3^)	258	19.03 ± 40.94	0.210	92.263
C_28_H_30_O_4_ (ng/m^3^)	258	18.73 ± 73.86	0.809	1085.231
C_15_H_15_F_5_O_4_ (ng/m^3^)	258	16.16 ± 123.64	0.611	1946.794
C_24_H_20_F_10_O_4_ (ng/m^3^)	258	15.37 ± 157.83	0.192	2534.171
C_14_H_24_O_3_Si_2_ (ng/m^3^)	258	14.35 ± 54.89	0.702	642.766
C_23_H_25_NO_6_ (ng/m^3^)	258	12.21 ± 8.28	1.601	22.628
C_14_H_22_O_2_ (ng/m^3^)	258	12.02 ± 31.85	1.861	415.086
C_16_H_11_NO_4_ (ng/m^3^)	258	11.85 ± 14.50	2.878	40.763
C_17_H_13_ClO_4_ (ng/m^3^)	258	8.88 ± 54.67	0.546	685.089
C_20_H_24_O_2_ (ng/m^3^)	258	6.88 ± 44.57	0.726	701.380
C_17_H_19_F_5_O_4_ (ng/m^3^)	258	6.64 ± 17.54	0.626	150.191

* This feature shares an identical molecular formula with the second-most abundant compound but is a distinct isomer.

## Data Availability

The data presented in this study are available upon request from the corresponding author. The data are not publicly available due to privacy restrictions.

## Supplementary Materials

The following supporting information can be downloaded at: https://www.mdpi.com/article/10.3390/metabo16080553/s1, Figure S1: Volcano Plot 2 of Plasma Metabolites Associated with Ester Exposure in PM_2.5_; Figure S2: Volcano Plot 3 of Plasma Metabolites Associated with Ester Exposure in PM_2.5_; Figure S3: Metabolic Pathway Enrichment Results for C23H28O6; Figure S4: Metabolic Pathway Enrichment Results for C16H20O6; Figure S5: Metabolic Pathway Enrichment Results for C16H11NO4; Figure S6: Metabolic Pathway Enrichment Results for C12H17NO2; Figure S7: Metabolic Pathway Enrichment Results for C23H25NO6; Table S1: Basic Characteristics of Individual PM_2.5_ bound OPEs and PAEs; Table S2: Compound identification and EI mass spectral similarity scores for PM_2.5_. Supplementary Methods: GC/MS Experimental Protocol, LC/MS Experimental Protocol, Metabolomic Data Analysis with MetaboAnalyst 5.0, Annotation Confidence Criteria.

## Abbreviations

The following abbreviations are used in this manuscript:

PM_2.5_	Ambient Fine Particulate Matter
GC–TOF or GC/MS	Gas Chromatograph Coupled With an Agilent Quadrupole Time-of-flight Mass Spectrometer
LC/MS	Liquid Chromatography–Mass Spectrometry
UV	Ultraviolet
CKD	Chronic Kidney Disease
RT	Retention Time
MSEA	Metabolite Set Enrichment Analysis
EF	Enrichment Factor
OPEs	Organophosphate Esters
PAEs	Phthalate Esters
BMI	Body Mass Index
CE	Cholesterol Esters
