# Supplementary material for: Untargeted Metabolomics Reveals Metabolic Perturbations in Community-Dwelling Elderly Exposed to PM2.5-Bound Ester Compounds
Source: Metabolites. 2026 Aug 5;16(8):553. doi: 10.3390/metabo16080553 (PMC13515499; doi:10.3390/metabo16080553)
Supplement: Supplementary file 1 [file metabolites-16-00553-s001.zip › Supplementary Methods.pdf]

GC/MS Experimental Protocol

I.PM$_{2.5}$ Filter Membrane Pretreatment Protocol

Quartz fiber filter membranes post-sampling were cut into small pieces and transferred into 15 mL centrifuge tubes. Add 2.5 mL of n-hexane and 2.5 mL of dichloromethane as the extraction solvent. Sonicate at 40°C for 20 min. Repeat the extraction process once by adding another 2.5 mL of n-hexane and 2.5 mL of dichloromethane, followed by sonication at 40°C for an additional 20 min. Combine the extracts and filter through a 0.22 μm hydrophilic syringe filter into a 10 mL centrifuge tube wrapped in tin foil. Lyophilize the filtrate until near dryness. Reconstitute the residue with a mixture of 17.5 μL of carbon tetrachloride and 250 μL of acetonitrile, and vortex thoroughly. Subsequently, add 2.5 mL of ultrapure water and rapidly mix to form an emulsion system. Allow the mixture to stand for 5 min. Centrifuge at 3500 rpm for 5 min, and collect the sediment phase for subsequent GC-MS analysis.

II.Data Acquisition and Processing

Data acquisition and processing for this study were performed using the following Agilent software packages:Agilent MassHunter Acquisition Software (B.07.02), Agilent MassHunter Qualitative Analysis Software, with PCDL Manager standalone tool (B.07.00), Agilent MassHunter Quantitative Analysis Software, with Library Editor and Unknown Analysis standalone tools (B.07.01), Agilent Mass Profiler Professional Software (Version 13.1).

III.GC/Q-TOF Instrument Parameters Used in This Experiment

Item	Parameter Setting
Gas Chromatography Conditions	
GC	Agilent 7890A
Injector	Multi-mode inlet (MMI)
Mode	Splitless
Septum purge flow rate	3 mL/min
Injector temperature program	70 °C (hold 0.2 min), then ramp to 325 °C at 600 °C/min (hold 7 min)
Liner	Ultra-high temperature splitless single-taper liner with wool (Part No. 5182-3163)
Carrier gas	Helium
Flow rate	1.3 mL/min (constant flow)
Split vent purge flow rate	60 mL/min (at 2.73 min)
Carrier gas saver mode	20 mL/min (at 3 min)
Oven temperature program	50 °C (hold 3 min), then ramp to 320 °C at 6 °C/min (hold 7 min)

Item	Parameter Setting
Equilibration time	1 min
Run time	55 min
Column	Agilent DB-5ms, 30 m × 250 μm, 0.25 μm (Part No. 122-5532)
Injection volume	2 μL
Mass Spectrometry Conditions	
MS	Agilent 7200
Tuning	Auto tune
Transfer line	280 °C
MS ion source (EI and CI)	300 °C
MS quadrupole	175 °C
Mass range	55–700 amu
Acquisition rate	5.00 spectra/s
Electron Impact Ionization	
EI emission current	35 μA
EI electron energy	70 eV
Chemical Ionization	
CI emission current	240 μA
CI gas flow rate	20% EPC
CI electron energy	115 eV
Mode	Positive ion
CI reagent gas	Methane
Collision cell EPC	Nitrogen, 1.5 mL/min

IV. Data Analysis

1.EI Source Data Analysis

Data files were processed using Agilent MassHunter Unknown Analysis software to perform deconvolution and matched against the NIST 2020 spectral library. A match score cutoff of > 70 was set to select the identified compounds.

2.MPP Analysis

The data were reprocessed using the Mass Profiler Professional (MPP) tool to perform deconvolution using precise mass numbers and the high-precision EI library, followed by spectrum and RT matching. This step facilitates the filtering of results to be exported to MPP software.

3.CI Data Analysis

Using the MassHunter Qualitative Analysis software, CI data were processed based on the "Molecular Formula" algorithm and possible adducts $[M+H]^+$, $[M-H]^-$, and $[M+CH_4]^+$ to analyze the CI data. The low-precision EI library was used as the molecular

formula database. In addition, the user-created extractables and non-extractables libraries were used to search for other identifiable compounds in the CI data.

4.Structural Analysis by CI/MS

Structural elucidation was performed using the Find by Targeted MS function in the MassHunter Qualitative Analysis software. The data were processed using ACD software (ACD Labs, Toronto) to confirm and visualize the fragment ion structures.

LC/MS Experimental Protocol

I. Objective

To quantify metabolites in plasma or urine samples.

II. Instruments, Reagents & Consumables

Major Reagents & Consumables: Acetonitrile (HPLC grade), formic acid (HPLC grade), ultrapure water, mass spectrometry tuning solution, liquid nitrogen, 0.22 μm syringe filters, 2 mL glass HPLC vials, HPLC columns.
Major Instruments: Automatic high-pressure steam sterilizer (G165TW), digital blast drying oven (GZX-9070MBE), electronic balance, Mini-4lc microcentrifuge, TD5A-WS benchtop low-speed centrifuge, H1650-W benchtop high-speed microcentrifuge, QT-1 vortex mixer, ultrasonic cleaner (KQ-500E), micropipettes.

III. Experimental Procedures

1. Plasma Sample Preparation:

Retrieve plasma samples from the -80°C freezer and thaw at room temperature for 3 hours. Transfer 150 μL of plasma into a numbered EP tube. Add 300 μL of acetonitrile, vortex for 30 seconds, and incubate at 4°C for 10 minutes. Subsequently, add another 150 μL of acetonitrile, vortex for 30 seconds, and store at 4°C for 3 hours to ensure complete protein precipitation. Centrifuge at 16,000 RCF for 30 minutes at 4°C. Collect 500 μL of the supernatant and filter through a 0.22 μm membrane into a numbered EP tube. Aliquot 100–150 μL into a 200 μL glass insert, place the insert into a 2 mL autosampler vial for analysis, and store the remaining sample at -80°C. Samples prepared on the same day must be analyzed on the same day. For quality control (QC), pool 10 μL from each sample into a separate tube.

2. Urine Sample Preparation:

Thaw samples at 4°C. Transfer 100 μL of urine into a 1.5 mL EP tube, add 100 μL of ultrapure water, and vortex for 5 minutes to ensure thorough mixing. Centrifuge at 21,000 × gfor 10 minutes. Filter the supernatant through a 0.22 μm membrane into a numbered EP tube to obtain the plasma samples. Transfer 200 μL into a glass insert. Prepare QC samples by pooling 10 μL from each sample into a tube, mixing thoroughly, transferring 100 μL into an EP tube, adding 100 μL of ultrapure water, and repeating the above centrifugation and filtration steps.

3. LC-MS Analysis (Identical for Urine and Plasma):

(1) Chromatographic Conditions

Column: Agilent SB-C18 column (2.1 × 50 mm, 1.8 μm); Column temperature: 37°C; Injection volume: 20 μL.

Mobile phase A: Ultrapure water with 0.1% formic acid; Mobile phase B: Acetonitrile with 0.1% formic acid. Flow rate: 0.25 mL/min. Prior to analysis, degas mobile phases using a KQ-250E ultrasonic cleaner to prevent air bubbles.
Linear gradient elution program (flow rate: 0.25 mL/min). Equilibrate the column for 30 min before each batch injection. Elute samples over 30 min from A:B (98:2) to A:B (0:100).

Insert an ultrapure water wash step after each sample. Details below:

Sample Gradient:

Time (min)	Flow Rate (mL/min)	A (%)	B (%)	Pressure (bar)
0.00	0.25	98.0	2.0	400.00
9.00	0.25	40.0	60.0	400.00
18.00	0.25	40.0	60.0	400.00
20.00	0.25	0.0	100.0	400.00
30.00	0.25	0.0	100.0	400.00

Water Wash Step:

Time (min)	Flow Rate (mL/min)	A (%)	B (%)	Pressure (bar)
7.00	0.25	2.0	98.0	400.00
14.00	0.25	98.0	2.0	400.00

(2) Mass Spectrometry Conditions

Instrument equipped with a TurboIonSpray (ESI) source and OpenLAB CDS data processing system. Detection performed in both positive and negative ion modes. Parameters as follows:

Parameter	Positive Mode	Negative Mode
Scan Mode	EMS	EMS
Ionspray Voltage (IS)	5500 V	-4500 V
Curtain Gas (CUR)	40 psi	30 psi
GS1	80 psi	80 psi
GS2	70 psi	70 psi
TEM	375 °C	375 °C
DP	40 V	-50 V
EP	10 V	-10 V
CE	10 V	-10 V

Scan speed: 4000 Da/s. All gases used were nitrogen. Mass scan range: m/z50–1500. Scan time: 0.3 s; Inter-scan delay: 0.02 s. Prior to analysis, calibrate the mass axis using the tuning solution.

Data acquisition and processing were performed using XCMS software. Raw data from the LC-MS platform was converted to ".mzXML" format using Agilent Mass Hunter software. Subsequent preprocessing, including peak picking, alignment, matching, and retention time correction, was conducted via XCMS Online.

IV. Quality Control

1.Strict adherence to experimental protocols is mandatory to ensure consistency. Prevent contamination and mislabeling. Avoid repeated freeze-thaw cycles of blood samples to prevent degradation. Sterilize all reagents and consumables using autoclaving where applicable.

2.During metabolite profiling, insert one QC sample after every 10 study samples. Prior to statistical analysis, normalize metabolite responses in each batch against the corresponding QC sample responses to mitigate batch effects.

Metabolomic Data Analysis with MetaboAnalyst 5.0

I.Background

The Pathway Analysis module combines results from powerful pathway enrichment analysis with pathway topology analysis to help researchers identify the most relevant pathways involved in the conditions under study.

There are many commercial pathway analysis software tools,such as Pathway Studio, MetaCore,or Ingenuity Pathway Analysis(IPA),etc.Compared to these commercial tools, the pathway analysis module was specifically developed for metabolomics studies. It uses high-quality KEGG metabolic pathways as the backend knowledgebase. This module integrates many well-established(i.e., univariate analysis, over-representation analysis)methods, as well as novel algorithms and concepts(i.e.,Global Test, GlobalAncova, network topology analysis into pathway analysis. Another feature is a Google-Map style interactive visualization system to deliver the analysis results in an intuitive manner.

II.Data Input

The Pathway Analysis module accepts either a list of compound labels(common names,HMDB IDs,or KEGG IDs)with one compound per row,or a compound concentration table with samples in rows and compounds in columns.The second column must be phenotype labels(binary,multi-group,or continuous).The table is uploaded as comma-separated values(.csv).

III.Compound Name Matching

The first step is to standardize the compound labels used in user-uploaded data.This is a necessary step since these compounds will be subsequently compared with compounds contained in the pathway library.There are three outcomes from the step—exact match,approximate match(for common names only),and no match.Users should click the textbfView button from the approximate matched results to manually select the correct one.Compounds without a match will be excluded from the subsequent pathway analysis.

IV.Pathway Analysis

In this step,users are asked to select a pathway library,as well as specify the algorithms for pathway enrichment analysis and pathway topology analysis.

4.1 Pathway Library

There are 15 pathway libraries currently supported,with a total of 1173 pathways:

- Homo sapiens(human)[]
- Mus musculus(mouse)[]
- Rattus norvegicus(rat)[]
- Bos taurus(cow)[]
- Danio rerio(zebrafish)[]
- Drosophila melanogaster(fruitfly)[]

●Caenorhabditis elegans(nematode)[]

●Saccharomyces cerevisiae(yeast)[]

●Oryza sativa japonica(Japanese rice)[]

●Arabidopsis thaliana(thale cress)[]

●Escherichia coli K-12 MG1655[]

●Bacillus subtilis[]

●Pseudomonas putida KT2440[]

●Staphylococcus aureus N315(MRSA/VSSA)[]

●Thermotoga maritima[]

Your selected pathway library code is hsa(KEGG organisms abbreviation).

4.2 Over Representation Analysis

Over-representation analysis tests whether a particular group of compounds is represented more than expected by chance within the user-uploaded compound list.In the context of pathway analysis,we are testing whether compounds involved in a particular pathway are enriched compared to random hits.MetPA offers two of the most commonly used methods for over-representation analysis:

●Fisher’s Exact test

●Hypergeometric Test

Please note,MetPA uses a one-tailed Fisher’s exact test,which will give essentially the same result as the result calculated by the hypergeometric test.

The selected over-representation analysis method is the Hypergeometric test.

4.3 Pathway Topology Analysis

The structure of biological pathways represents our knowledge about the complex relationships among molecules within a cell or a living organism.However,most pathway analysis algorithms fail to take structural information into consideration when estimating which pathways are significantly changed under conditions of study.It is well-known that changes in more important positions of a network will trigger a more severe impact on the pathway than changes occurring in marginal or relatively isolated positions.

The pathway topology analysis uses two well-established node centrality measures to estimate node importance:degree centrality and betweenness centrality.Degree centrality is defined as the number of links occurring at a node.For a directed graph,there are two types of degree:in-degree for links coming from other nodes,and out-degree for links initiated from the current node.Metabolic networks are directed graphs.Here,we only consider the out-degree for the node importance measure.It is assumed that nodes upstream will have regulatory roles for the downstream nodes,not vice versa.The betweenness centrality measures the number of shortest paths going through the node.Since the metabolic network is directed,we use the relative betweenness centrality for a metabolite as the importance measure.The degree centrality measure focuses more on local connectivities,while the betweenness centrality measure focuses more on global network topology.Please note,for comparison among different pathways,the node importance values calculated from centrality measures are further normalized by the sum of the importance of the pathway.Therefore,the total/maximum importance of each pathway is 1;the importance measure of each metabolite node is actually the percentage w.r.t the total pathway importance,and the pathway impact value is the cumulative

percentage from the matched metabolite nodes.

Your selected node importance measure for topological analysis is relative betweenness centrality.

Annotation Confidence Criteria

Compound annotations in this study were assigned confidence levels following the framework of Schymanski et al. (2014), adapted to the specific workflow of non-targeted GC-EI-MS screening using Agilent MassHunter Qualitative Analysis (v10.0) and the NIST20 mass spectral library. No annotations met the criteria for Confidence Level 1 (CL1, confirmed structure), as authentic reference standards for the vast majority of detected $PM_{2.5}$-bound ester compounds are not commercially available, and no orthogonal validation (e.g., co-analysis of reference standards under identical instrumental conditions) was performed. All reported assignments are therefore tentative annotations at CL2 or lower, as detailed below.

Confidence Level 1 (CL1, Confirmed Identification)

Confirmed structure represents the ideal situation, where the proposed structure has been confirmed via appropriate measurement of a reference standard with MS, MS/MS and retention time matching. If possible, an orthogonal method should also be used.

Confidence Level 2 (CL2, Putative Annotation)

CL2 indicates a probable structure supported by reproducible experimental evidence, subdivided into two subcategories based on the source of supporting data:

CL2a (Library match):Assignments where the detected mass spectrum yielded a NIST20 library match score ≥70%, consistent with the screening threshold recommended by Xu et al. for GC-MS non-targeted environmental screening. The MassHunter match score integrates concordance of fragment ion m/zvalues, relative ion abundances, and overall spectral pattern; a score ≥70% indicates high similarity to a reference entry in the NIST20 library. However, this threshold serves exclusively as a screening criterion for tentative annotation, not a criterion for definitive structural identification. Differences in instrument parameters (e.g., ion source temperature, electron energy, quadrupole tuning) between the NIST reference spectrum and the current analysis, as well as potential isomeric interferences or in-source thermal degradation artifacts, preclude confirmation without authentic standards.

CL2b (Diagnostic fragmentation):Assignments where no NIST20 match ≥70% was obtained, but the observed fragmentation pattern contained diagnostic ions characteristic of a known compound class, combined with plausible atmospheric occurrence. For example, cholesteryl derivatives were annotated based on diagnostic fragment ions at m/z329, 368, and 386, consistent with the sterol backbone structure.

All CL2 annotations were manually curated to exclude features with evident retention time drift or anomalous ion ratios, and remain subject to future validation with authentic standards.

Confidence Level 3 (CL3, Tentative Candidate)

CL3 denotes cases where partial evidence supports possible structures but is insufficient to assign a single exact structure. Typical scenarios in this study included:

NIST20 match scores between 50–69% with a plausible molecular formula derived

from fragment ion combinations;

Clear molecular ion peaks with isotopic patterns consistent with a candidate formula, but uninformative fragmentation preventing resolution of structural isomers (e.g., positional isomers of long-chain alkyl esters).

Confidence Levels 4–5 (Limited Structural Information)

CL4 (Unequivocal molecular formula): Rarely assigned, reserved for cases where a molecular formula could be unambiguously derived from isotopic abundance ratios (e.g., for halogenated compounds) despite uninformative or absent fragmentation.

CL5 (Feature of interest): Applied to a small number of chromatographic features with unique mass spectral signatures relevant to $PM_{2.5}$ exposure assessment, but no assignable formula or structural information. Blank sample controls were used to confirm these features were not derived from sample preparation or instrument background.

Reporting Conventions

All incorporated compounds met the CL2a criteria, defined by a NIST20 library match score ≥70%.
