# Supplementary material for: Untargeted Metabolomics Reveals Metabolic Perturbations in Community-Dwelling Elderly Exposed to PM2.5-Bound Ester Compounds
Source: Metabolites. 2026 Aug 5;16(8):553. doi: 10.3390/metabo16080553 (PMC13515499; doi:10.3390/metabo16080553)
Supplement: Supplementary file 1 [file metabolites-16-00553-s001.zip › Supplementary Tables and Figures.pdf]

Table S1. Basic Characteristics of Individual $PM_{2.5}$ bound OPEs and PAEs

Organophosphate esters				
$C_5H_{12}FO_2P$（ng/m³）	258	5.33 ± 0.47	5.288	10.575
$C_{15}H_{29}N_2O_6P$（ng/m³）	258	1.52 ± 2.99	0.076	14.248
$C_{12}H_{29}O_3Psi$（ng/m³）	258	1.21 ± 0.11	1.205	2.41
$C_3H_8FO_3P$（ng/m³）	258	0.99 ± 0.09	0.983	1.966
$C_{15}H_{24}F_3O_6P$（ng/m³）	258	0.97 ± 0.39	0.914	4.284
$C_7H_{16}FO_3P$（ng/m³）	258	0.96 ± 0.08	0.949	1.897
$C_{23}H_{49}O_4P$（ng/m³）	258	0.89 ± 0.65	0.783	6.336
$C_{14}H_{35}O_3PSi_2$（ng/m³）	258	0.77 ± 1.12	0.382	7.349
$C_{10}H_{22}FO_3P$（ng/m³）	258	0.74 ± 0.06	0.735	1.47
$C_{10}H_{22}FO_3P$（ng/m³）	258	0.72 ± 0.06	0.711	1.421
$C_{15}H_{31}O_4P$（ng/m³）	258	0.64 ± 0.06	0.636	1.272
$C_{14}H_{31}O_4P$（ng/m³）	258	0.59 ± 0.05	0.587	1.174
$C_{25}H_{43}O_3P$（ng/m³）	258	0.49 ± 0.04	0.49	0.98
$C_9H_{21}O_4P$（ng/m³）	258	0.46 ± 2.81	0.011	31.241
$C_{19}H_{41}O_4P$（ng/m³）	258	0.41 ± 0.04	0.404	0.808
$C_7H_{17}O_4P$（ng/m³）	258	0.40 ± 0.37	0.316	2.745
$C_9H_{11}Cl_2O_3PS$（ng/m³）	258	0.40 ± 0.63	0.311	6.344
$C_9H_{18}Cl_3O_4P$（ng/m³）	258	0.39 ± 0.43	0.083	2.405
$C_6H_{15}O_4P$（ng/m³）	258	0.34 ± 0.08	0.324	1.04
$C_{15}H_{33}O_4P$（ng/m³）	258	0.32 ± 2.73	0.077	31.096
Phthalate esters				
$C_{12}H_{14}O_6$（ng/m³）	258	4.01 ± 25.10	0.074	174.163
$C_{14}H_{18}O_6$（ng/m³）	258	3.30 ± 4.25	0.257	24.921
$C_{26}H_{26}O_4$（ng/m³）	258	3.14 ± 3.27	0.199	22.464
$C_{18}H_{28}O_4$（ng/m³）	258	2.50 ± 0.22	2.477	4.954
$C_{15}H_{10}FNO_6$（ng/m³）	258	2.38 ± 3.35	0.511	14.34
$C_{10}H_{10}O_4$（ng/m³）	258	2.27 ± 2.19	1.872	24.451
$C_{16}H_{13}ClO_4$（ng/m³）	258	2.27 ± 0.20	2.25	4.499
$C_{24}H_{22}O_4$（ng/m³）	258	2.24 ± 3.73	0.102	17.583
$C_{17}H_{16}O_4$（ng/m³）	258	2.08 ± 0.18	2.059	4.118
$C_{14}H_{18}O_6$（ng/m³）	258	1.96 ± 5.07	0.015	40.745
$C_{19}H_{20}O_5$（ng/m³）	258	1.92 ± 0.17	1.906	3.812
$C_{32}H_{22}F_4O_4$（ng/m³）	258	1.91 ± 0.17	1.895	3.789
$C_{22}H_{34}O_4$（ng/m³）	258	1.83 ± 0.16	1.812	3.625
$C_{18}H_{13}F_5O_4$（ng/m³）	258	1.74 ± 2.03	0.185	12.557
$C_{26}H_{50}O_4$（ng/m³）	258	1.64 ± 0.14	1.63	3.26
$C_{20}H_{22}O_4$（ng/m³）	258	1.54 ± 0.13	1.533	3.065
$C_{16}H_{22}O_4$（ng/m³）	258	1.35 ± 5.83	0.059	50.408
$C_{16}H_{11}F_5O_4$（ng/m³）	258	1.23 ± 0.11	1.216	2.431
$C_{24}H_{28}O_6$（ng/m³）	258	1.14 ± 0.10	1.126	2.253
$C_{18}H_{26}O_4$（ng/m³）	258	1.12 ± 0.70	0.996	6.93

Table S2. Compound identification and EI mass spectral similarity scores for $PM_{2.5}$

Detected Formula	CAS[1]	Annotated Compound Name[1]	MassHunter matching scores
$C_{28}H_{46}O_2$	4351-55-7	Cholesteryl formate	82
$C_{13}H_{18}O_3$	54889-98-4	Ethyl benzoate	81
$C_{23}H_{28}O_6$	1000358-58-3	2,2-bis-benzyloxymethyl-malonic acid diethyl ester	76
$C_{16}H_{20}O_6$	56704-62-2	Benzylmalonate diester	76
$C_{12}H_{17}NO_2$	1000331-48-4	4-picolinic acid methyl ester	82
$C_{17}H_{16}O_3Si$	1000458-48-7	Trimethylsilyl benzoate	73
$C_{13}H_{18}O_3$*	1000331-30-9	Isobutyl benzoate	75
$C_{18}H_{17}ClO_5$	1000390-98-6	Chlorinated diphenyl ether carboxylate	81
$C_{25}H_{22}OS_2$	1000287-50-5	Diphenyl sulfide dicarboxylate	77
$C_8H_{14}O_2$	13170-00-8	Ethyl propionate	81
$C_{28}H_{30}O_4$	53417-65-5	Di-tert-butyl 3,3'-(anthracene-9,10-diyl)-diacrylate	83
$C_{15}H_{15}F_5O_4$	1000359-06-6	Pentafluorophenyl pentanoate	72
$C_{24}H_{20}F_{10}O_4$	1000365-32-0	Decafluorobiphenyl dicarboxylate	73
$C_{14}H_{24}O_3Si_2$	1000423-36-2	Bis(trimethylsilyl) adipate	78
$C_{23}H_{25}NO_6$	1000287-15-2	N-benzoyl-n-methylamino malonic acid dimethyl ester	85
$C_{14}H_{22}O_2$	24556-16-9	Isopropyl benzoate	81
$C_{16}H_{11}NO_4$	76644-24-1	1-phenanthrenecarboxylicacid, 10-nitro-, methyl ester	82
$C_{17}H_{13}ClO_4$	1000215-35-2	4-chlorobiphenyl-4-yl acetate	72
$C_{20}H_{24}O_2$	1000354-32-1	Butyl phenylacetate	81
$C_{17}H_{19}F_5O_4$	1000392-10-7	Pentafluorophenyl heptanoate	84
$C_5H_{12}FO_2P$	1189-87-3	Fluoromethyl methylphosphonate	77
$C_{15}H_{29}N_2O_6P$	1000192-35-6	Phosphoramidate triester	81
$C_{12}H_{29}O_3Psi$	1000383-80-7	Trimethylsilyl dodecanoate	82
$C_3H_8FO_3P$	352-52-3	Fluoropropyl phosphate	81
$C_{15}H_{24}F_3O_6P$	1000273-30-1	Trifluoromethyl phosphotriester	75
$C_7H_{16}FO_3P$	1000383-81-8	Fluoroheptyl phosphate	72
$C_{23}H_{49}O_4P$	1000273-31-2	Phosphoric acid tetracosyl ester	82
$C_{14}H_{35}O_3PSi_2$	1000273-30-1	Bis(trimethylsilyl) tridecyl phosphate	77
$C_{10}H_{22}FO_3P$	1000273-32-3	Fluorodecyl phosphate	79
$C_{13}H_{19}O_5P$	1000383-82-9	3-diethoxyphosphoryl-benzoic acid ethyl ester	79
$C_{15}H_{31}O_4P$	20195-08-8	Phosphoric acid pentadecyl ester	81
$C_{14}H_{31}O_4P$	1000273-33-4	Phosphoric acid tetradecyl ester	84

Detected Formula	CAS[1]	Annotated Compound Name[1]	MassHunter matching scores
$C_{25}H_{43}O_3P$	1000383-83-0	Phosphoric acid hexacosyl ester	71
$C_9H_{21}O_4P$	20195-08-8	Phosphoric acid nonyl ester	79
$C_{19}H_{41}O_4P$	1000273-34-5	Phosphoric acid nonadecyl ester	75
$C_7H_{17}O_4P$	1000273-35-6	Phosphoric acid heptyl ester	80
$C_9H_{11}Cl_2O_3PS$	1000273-36-7	Dichlorophenyl thiophosphate	83
$C_9H_{18}Cl_3O_4P$	13674-84-5	Trichlorononyl phosphate	73
$C_6H_{15}O_4P$	78-40-0	Phosphoric acid hexyl ester	73
$C_{15}H_{33}O_4P$	2528-38-3	Phosphoric acid pentadecyl ester	82
$C_{12}H_{14}O_6$	959-26-2	Propyl gallate	77
$C_{14}H_{18}O_6$	959-26-2	Butyl gallate	82
$C_{26}H_{26}O_4$	1000345-86-7	Diaryl ester	81
$C_{18}H_{28}O_4$	1000356-20-3	Diisobutyl phthalate	72
$C_{15}H_{10}FNO_6$	1000344-39-4	Nitrofluorophenyl phthalate	82
$C_{10}H_{10}O_4$	1000315-63-4	Dimethyl phthalate	82
$C_{16}H_{13}ClO_4$	131-11-3	Chlorodiphenyl carboxylate	83
$C_{24}H_{22}O_4$	1000315-71-6	Diaryl ester	85
$C_{17}H_{16}O_4$	1000357-09-2	Diphenyl dicarboxylate	85
$C_{14}H_{18}O_6$	1000315-67-5	Butyl gallate	85
$C_{19}H_{20}O_5$	1000323-88-7	Hydroxybutyl benzoate	80
$C_{32}H_{22}F_4O_4$	1000344-40-7	Tetrafluorodiphenyl dicarboxylate	81
$C_{22}H_{34}O_4$	1000357-10-5	Diisohexyl phthalate	85
$C_{18}H_{13}F_5O_4$	1000323-89-8	Pentafluorophenyl benzoate	80
$C_{26}H_{50}O_4$	1000356-19-0	Diisodecyl phthalate	79
$C_{20}H_{22}O_4$	1000315-48-7	Diphenyl butyrate	85
$C_{16}H_{22}O_4$	1000315-66-4	Dibutyl phthalate	79
$C_{16}H_{11}F_5O_4$	84-74-2	Pentafluorophenyl naphthoate	85
$C_{24}H_{28}O_6$	1000323-90-1	Bis(2-ethylhexyl) phthalate	75
$C_{18}H_{26}O_4$	1000344-41-8	Diisooctyl phthalate	75

1 Tentative IDs (names/CAS) from NIST20/MassHunter. GC-MS reliably yields molecular formulas, peak intensities (basis for semi-quantitative concentration derivation), and diagnostic ester fragments; exact chemical entities remain to be validated.

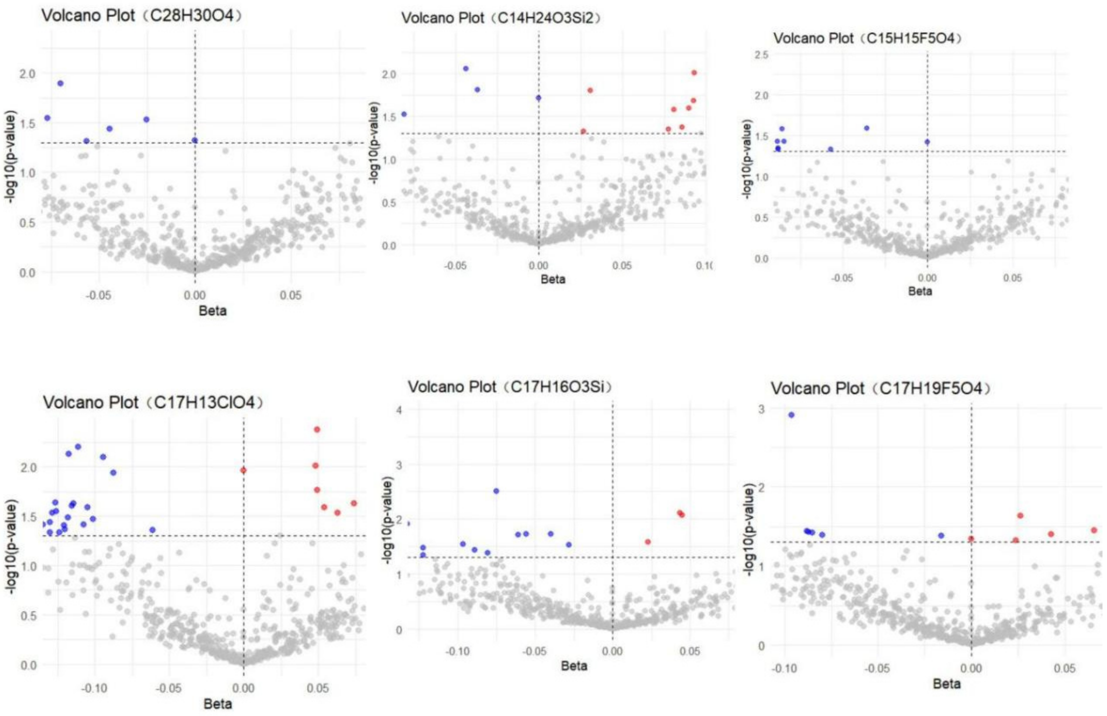

Figure S1. Volcano Plot 2 of Plasma Metabolites Associated with Ester Exposure in $PM_{2.5}$ (6 **Metabolites)**

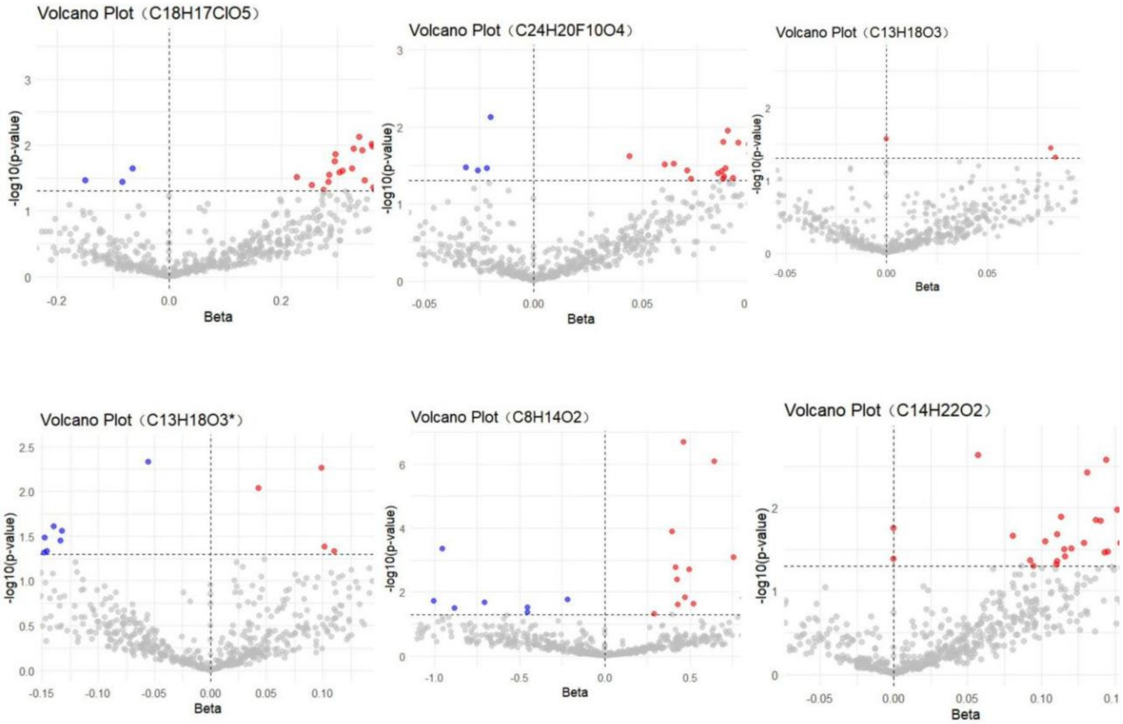

Figure S2. Volcano Plot 3 of Plasma Metabolites Associated with Ester Exposure in $PM_{2.5}$ (6 Metabolites)

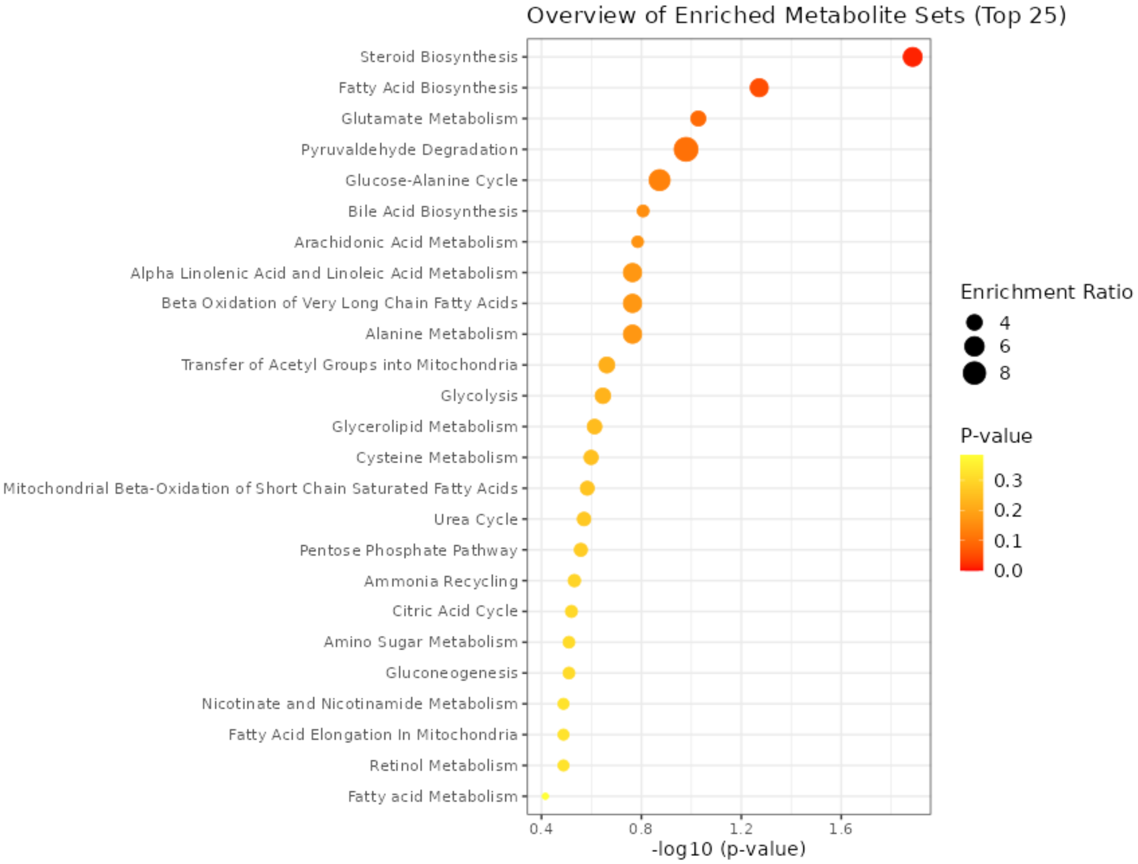

Figure S3. Metabolic Pathway Enrichment Results for C23H28O6

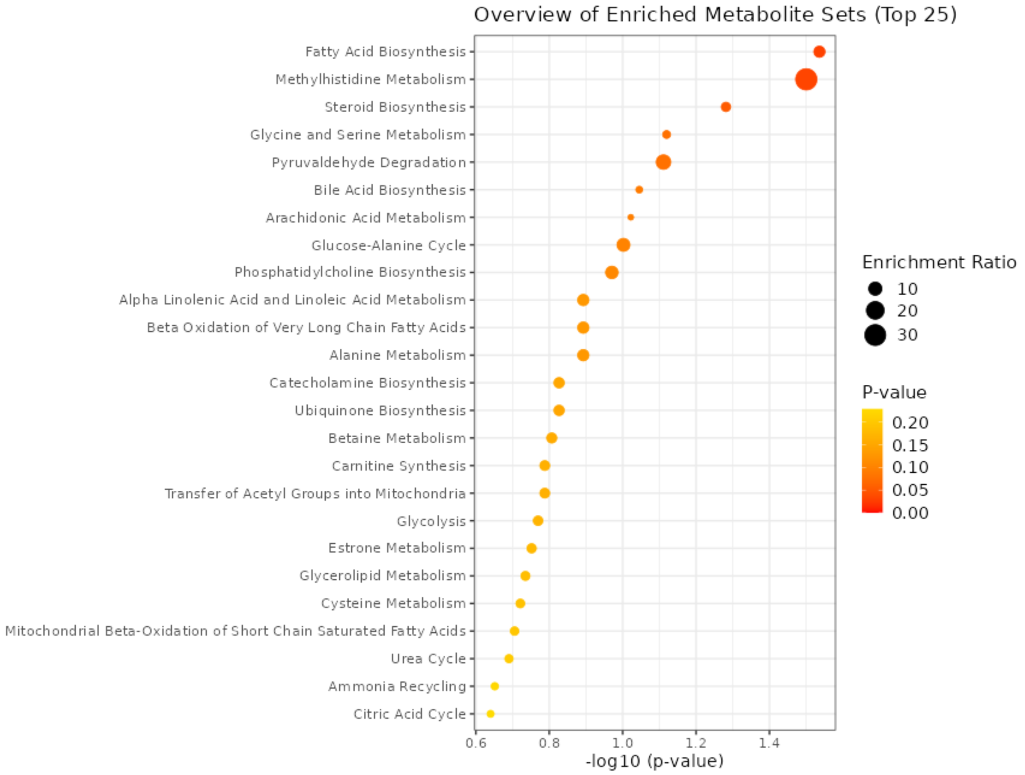

Figure S4. Metabolic Pathway Enrichment Results for C16H20O6

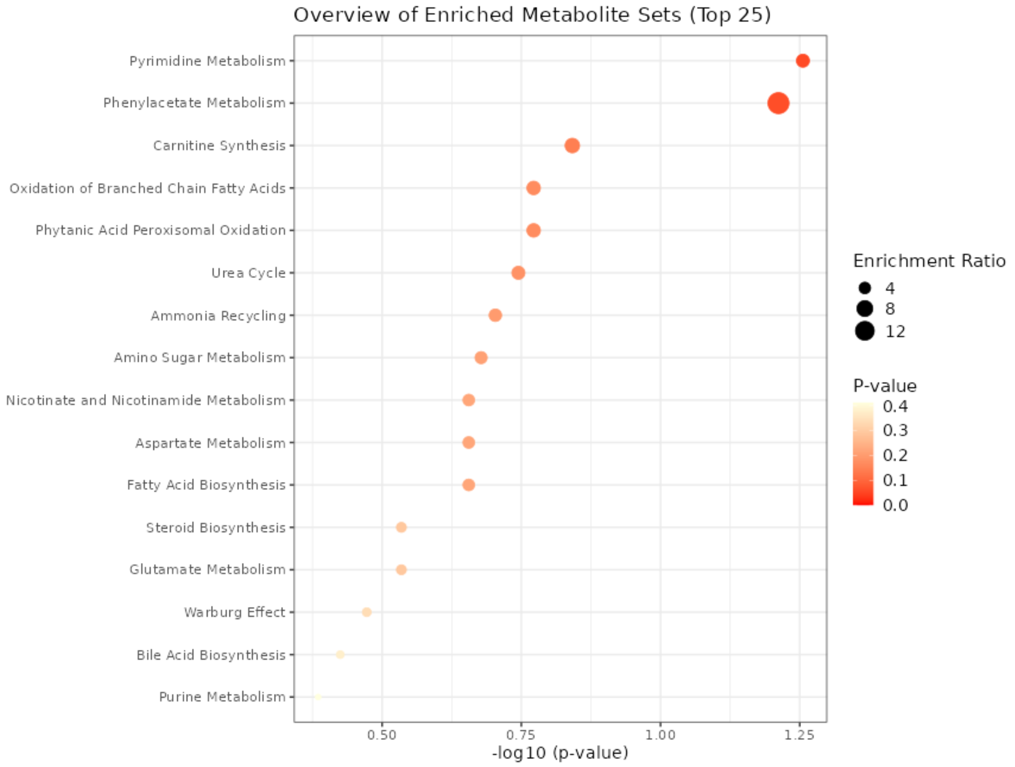

Figure S5. Metabolic Pathway Enrichment Results for C16H11NO4

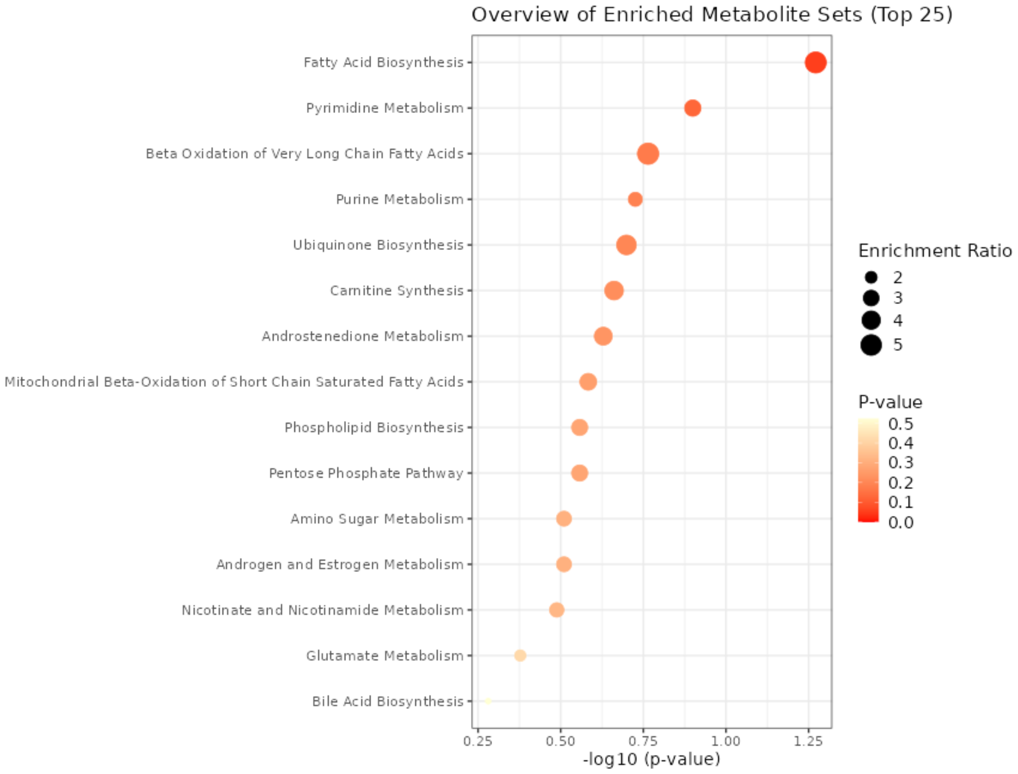

Figure S6. Metabolic Pathway Enrichment Results for C12H17NO2

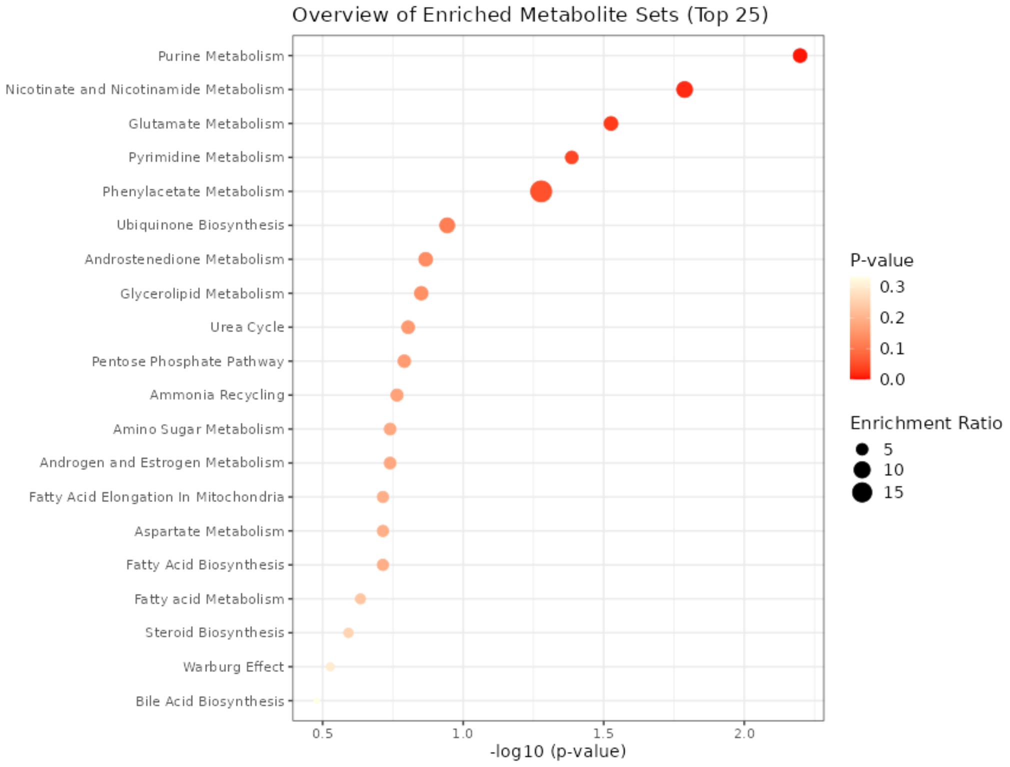

Figure S7. Metabolic Pathway Enrichment Results for C23H25NO6
